# Genetic and Epigenetic Factors at *COL2A1* and *ABCA4* Influence Clinical Outcome in Congenital Toxoplasmosis

**DOI:** 10.1371/journal.pone.0002285

**Published:** 2008-06-04

**Authors:** Sarra E. Jamieson, Lee-Anne de Roubaix, Mario Cortina-Borja, Hooi Kuan Tan, Ernest J. Mui, Heather J. Cordell, Michael J. Kirisits, E. Nancy Miller, Christopher S. Peacock, Aubrey C. Hargrave, Jessica J. Coyne, Kenneth Boyer, Marie-Hélène Bessieres, Wilma Buffolano, Nicole Ferret, Jacqueline Franck, François Kieffer, Paul Meier, Dorota E. Nowakowska, Malgorzata Paul, François Peyron, Babill Stray-Pedersen, Andrea-Romana Prusa, Philippe Thulliez, Martine Wallon, Eskild Petersen, Rima McLeod, Ruth E. Gilbert, Jenefer M. Blackwell

**Affiliations:** 1 Cambridge Institute for Medical Research and Department of Medicine, University of Cambridge School of Clinical Medicine, Addenbrookes Hospital, Cambridge, United Kingdom; 2 Centre for Paediatric Epidemiology and Biostatistics, Institute of Child Health, University College London, London, United Kingdom; 3 Departments of Ophthalmology, Medicine, Pediatrics, Committees on Immunology, Molecular Medicine, and Genetics, University of Chicago, and Michael Reese Hospital and Medical Center, Chicago, Illinois, United States of America; 4 Institute of Human Genetics, Newcastle University, International Centre for Life, Newcastle upon Tyne, United Kingdom; 5 Department of Pediatrics, Division of Pediatric Infectious Diseases, Rush University Medical Center, Chicago, Illinois, United States of America; 6 Service de Parasitologie et Mycologie, CHU Rangueil, Toulouse, France; 7 Department of Paediatrics, University of Naples "Frederico II", Naples, Italy; 8 Service de Parasitologie et Mycologie, Hopital Archet II, Nice, France; 9 Service de Parasitologie, CHU de la Timone, Marseille, France; 10 Department of Paediatrics, Institut de Puériculture, Paris, France; 11 Department of Biostatistics, Columbia University, New York, New York, United States of America; 12 Department of Fetal-Maternal Medicine and Gynecology, Medical University, Lodz, Rzgowska, Poland; 13 Department and Clinic of Tropical and Parasitic Diseases, University of Medical Sciences, Poznań, Poland; 14 Hospices Civils de Lyon, Service de Parasitologie, Hôpital de la Croix-Rousse, Lyon, France; 15 Department of Obstetrics and Gynaecology, University of Oslo, Rikshospitalet-Radiumhospitalet, Sognsvannsvn, Oslo, Norway; 16 Department of Pediatrics, Division of Neonatology, Congenital Disorders and Intensive Care, Medical University of Vienna, Vienna, Austria; 17 Toxoplasmosis Laboratory, Institut de Puériculture, Paris, France; 18 Department of Infectious Diseases, Aarhus University Hospital, Aarhus, Denmark; Ohio State University Medical Center, United States of America

## Abstract

**Background:**

Primary *Toxoplasma gondii* infection during pregnancy can be transmitted to the fetus. At birth, infected infants may have intracranial calcification, hydrocephalus, and retinochoroiditis, and new ocular lesions can occur at any age after birth. Not all children who acquire infection *in utero* develop these clinical signs of disease. Whilst severity of disease is influenced by trimester in which infection is acquired by the mother, other factors including genetic predisposition may contribute.

**Methods and Findings:**

In 457 mother-child pairs from Europe, and 149 child/parent trios from North America, we show that ocular and brain disease in congenital toxoplasmosis associate with polymorphisms in *ABCA4* encoding ATP-binding cassette transporter, subfamily A, member 4. Polymorphisms at *COL2A1* encoding type II collagen associate only with ocular disease. Both loci showed unusual inheritance patterns for the disease allele when comparing outcomes in heterozygous affected children with outcomes in affected children of heterozygous mothers. Modeling suggested either an effect of mother's genotype, or parent-of-origin effects. Experimental studies showed that both *ABCA4* and *COL2A1* show isoform-specific epigenetic modifications consistent with imprinting.

**Conclusions:**

These associations between clinical outcomes of congenital toxoplasmosis and polymorphisms at *ABCA4* and *COL2A1* provide novel insight into the molecular pathways that can be affected by congenital infection with this parasite.

## Introduction

*Toxoplasma gondii* is a ubiquitous protozoan parasitic infection that, if acquired for the first time during pregnancy, can be transmitted to the fetus. At birth, infants infected *in utero* may have intracranial calcification, hydrocephalus, and ocular disease broadly defined as retinochoroiditis or inflammation of the retina and choroid with associated vitritis [1]–[3]. New ocular lesions can occur at any age after birth, in untreated and some treated children. Whilst severity of disease is influenced by trimester in which infection is acquired by the mother [4], [5], other factors including genetic predisposition may contribute. For example, previous studies suggest that genes affecting immune response, including HLA [6], influence clinical outcome in the child. However, since infants who have the most severe clinical signs in the brain and eye are those infected early in pregnancy [4], [5] when fetal immunity is least well developed, we considered whether genes that encode molecules that play a role in developmental processes could contribute to clinical phenotype observed in the child. This could provide unique insight into events *in utero* and post-natally that determine the clinical outcome of infection. In particular, we hypothesized that propensity for *T. gondii* to cause eye disease may be associated with genes previously implicated in congenital or juvenile onset ocular disease. Two genes were of specific interest, *ABCA4* encoding ATP-binding cassette transporter subfamily A member 4 associated with juvenile onset retinal dystrophies including Stargardt's disease [7], [8], and *COL2A1* encoding type II collagen associated with Stickler syndrome [9] in which there is congenital abnormal vitreous and lattice retinal degeneration. Although risk of transmission of the parasite to the fetus in pregnant women with primary infection ranges from <1–100% depending on the time in gestation when infection is acquired [5], incidence rates of clinical congenital toxoplasmosis, together with the fact that it is not a reportable disease, have precluded accumulation of large cohorts for genetic studies. Nevertheless, two unique cohorts have recently become available to test this specific genetic hypothesis. These cohorts are from the European Multicentre Cohort Study on Congenital Toxoplasmosis (EMSCOT) which recruited prospectively for mothers with primary *T. gondii* in pregnancy [1], [2], and from the National Collaborative Chicago-based Congenital Toxoplasmosis Study (NCCCTS) [3] in North America to which infants and children with congenital infection with *T. gondii* are referred. Using these cohorts we show that ocular and brain disease in congenital toxoplasmosis associate with polymorphisms in *ABCA4*, while polymorphisms at *COL2A1* encoding type II collagen associate only with ocular disease. Both loci show unusual inheritance patterns for the disease allele when comparing outcomes in heterozygous affected children with outcomes in affected children of heterozygous mothers, and modeling suggests either an effect of mother's genotype or parent-of-origin effects. The latter is supported by experimental data showing that both *ABCA4* and *COL2A1* show isoform-specific epigenetic modifications consistent with imprinting.

## Methods

### European study population

The European study was undertaken as an adjunct to European Multicentre Study of Congenital Toxoplasmosis (EMSCOT), referred to as GENET-EMSCOT. Ethical approvals for GENET-EMSCOT were obtained through the local ethical review boards of participating centres across Europe, and for the study as a whole from the Research Ethics Committee for Great Ormond Street Hospital and the Institute for Child Health in London. Screening, treatment and follow up schedules have been described in detail elsewhere [1]. For this study, mother-child pairs were eligible for inclusion if the child had congenital toxoplasmosis or, if the child was uninfected, the mother had evidence of seroconversion (i.e. change from IgG negative to IgG positive for specific antibodies to *T. gondii*) during pregnancy. Infected children were identified in centres that utilized universal prenatal or neonatal screening (Copenhagen, Stockholm, Lodz and Poznan). Uninfected children were identified only in those centres that utilized prenatal screening (Lyon, Paris, Marseille, Toulouse, Nice, Grenoble, Vienna, Naples, Oslo). Families were invited to participate by the clinician responsible for follow up after diagnosis of toxoplasmosis. The clinician recorded details reflecting the timing of maternal seroconversion, and in the child, confirmation of congenital infection status, detection of intracranial lesions based on cranial ultrasound after birth, and the age at detection of any retinochoroidal lesions or the age at the last negative ophthalmoscopy examination. We estimate that 85% of the mothers in the EMSCOT study had been treated prenatally [2]. Recent analyses of EMSCOT and other cohort studies provides no evidence for a statistically significant effect of prenatal treatment on brain or eye lesions [10]–[12]. Hence, although there is variability in prenatal treatment across the EMSCOT cohort, it is unlikely that this will confound the genetic statistical analysis performed here. To increase the power of the study, clinicians were asked to selectively recruit infected and affected children. For some centres (Lyon, Toulouse, Marseille, Paris, Lodz) this included prospective sampling and retrospective samples of stored sera or plasma from mother-child pairs with confirmed clinical congenital toxoplasmosis, and for whom comparable data were available to accurately complete questionnaires developed for the GENET-EMSCOT study. Within these groups, the study population was assumed to be representative of mothers and children identified by universal screening for toxoplasmosis. However, this could not be checked as families not enrolled were not recorded. The child's grandparents' countries of birth were recorded to provide an acceptable proxy for ethnicity. Buccal swabs from the mother and child were taken into transport/lysis buffer (10mmol/L TRIS base, 10mmol/L EDTA, 0.5% Na sarkosyl) and kept at ambient temperatures during postage to the laboratory in Cambridge. Plasma or serum samples stored at −20°C were shipped frozen to Cambridge. A total of 457 mother-child pairs met the inclusion criteria to participate in the study, which included successful preparation of DNA.

### North American Study Population

Case-parent trios for the North American cohort were from the National Collaborative Chicago-based Congenital Toxoplasmosis Study (NCCCTS) [3]. Ethical approval for the study was obtained from the local Institutional Review Boards of the University of Chicago and Michael Reese Hospital and Medical Center, and oversight was provided by an Internal Data Safety Monitoring Committee, the Data Safety Monitoring Board, and NIH. The diagnosis of congenital toxoplasmosis was confirmed on the basis of clinical findings and testing in the Toxoplasmosis Serology Laboratory (Palo Alto Medical Research Institute) as described [3], [5]. At birth or time of diagnosis, each child was examined in the same center in Chicago with standardized ophthalmologic examination and review of all medical records and a brain CT scan [3]. Samples for 176 clinically confirmed children were available for the genetic study, 138 from an ongoing treatment trial [3], [5], [13]. Inclusion criteria for these 138 children were as follows: (1) age less than 2.5 months at diagnosis, (2) diagnosis of congenital toxoplasmosis highly likely as previously described [13], (3) willingness to be periodically evaluated in Chicago, and (4) no concomitant immunosuppressive conditions. The additional 38 children presented after the first year of life and were therefore not treated during this time. However, their clinical evaluation was as described before [5]. Peripheral blood cells were isolated and cryopreserved from all children and their mothers and some fathers. A small sample (10 µl) of these cells in cryopreservation mix was placed in 100 µl transport/lysis buffer (as above), and shipped to Cambridge at ambient temperature. A total of 149 children and available parents met the inclusion criteria to participate in the study, which included successful preparation of DNA.

### Genotyping

**Figure 1:**
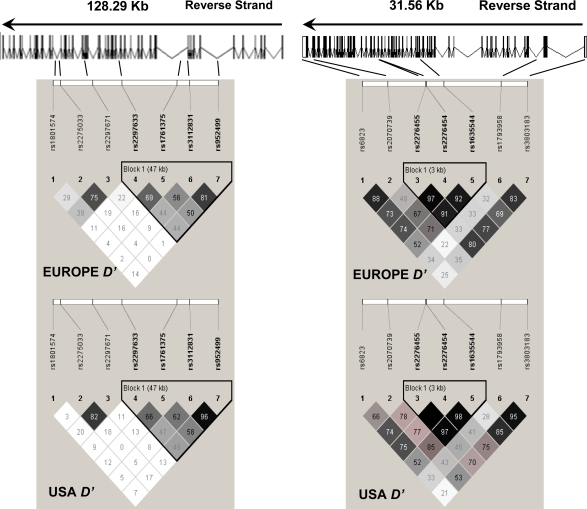
Gene structure and linkage disequilibrium plots for *ABCA4* (left panel) and *COL2A1* (right panel). Upper diagrams show positions of SNPs genotyped in relative to intron/exon structure of the gene. Lower diagrams show the linkage disequilibrium (*D′*) plots generated in Haploview (http://www.hapmap.org/) using data for each gene from the EMSCOT or NCCCTS cohorts as indicated. Linkage disequilibrium values (×100) between markers are indicated at the intercept of the two markers on the matrix. Where there is no value, D′ = 1 (i.e. 100). Haplotype blocks within each gene are outlined within the black triangles. The black (high) through grey to white (low) shading indicates the degree of confidence in the estimate of linkage disequilibrium between the markers. For the EMSCOT cohort, stepwise logistic regression analysis [50] of associations observed in the mothers (Online Supplementary Material, Text S1 plus Table S5) indicate that all of the association at *ABCA4* is accounted for by rs2997633 and rs1761375, implying that a single etiological variant in strong linkage disequilibrium with these two markers may account for the association. At *COL2A1*, SNPs rs2276455, rs1635544 and rs3803183 all add significant main effects when compared to rs2070739, but rs2070739 does not add significant main effects to any one of these markers. SNPs rs2276455, rs1635544 and rs3803183 do not add significant main effects to each other. Once pairs of these markers are taken into the model, the third SNP does not add significant main effects. A single etiological variant in this haplotype block could account for the association with *COL2A1*. Neither SNP (rs2070739, rs3803183) that results in a non-synonymous amino acid substitution appears to be the primary functional variant.

DNA was obtained from all samples by whole genome amplification of DNA extracted from the buccal swab buffer, or by direct amplification from the buccal buffer, serum, plasma, or lysis buffer from cells, using multiple displacement amplification (MDA, Molecular Staging, USA; now supplied by Qiagen). Over a number of large family-based studies undertaken in our laboratory we have demonstrated a rate of 1.1% to 2.6% allele drop out using DNA amplified in this way. Seven single nucleotide polymorphisms (SNPs) at *ABCA4* (rs1801574, rs2275033, rs2297671, rs2297633, rs176375, rs3112831, rs952499) and 7 SNPs at *COL2A1* (rs6823, rs2070739, rs2276455, rs2276454, rs1635544, rs1793958, rs3803183) were genotyped in both cohorts. We also examined 2 SNPs at *VMD2* encoding bestrophin 1 or vitelliform macular dystrophy protein 2, mutations in which are associated with an autosomal dominant juvenile-onset macular dystrophy [14] but the clinical phenotype differs from congenital toxoplasmosis, and 3 SNPs at *TIMP3* which encodes tissue inhibitor of metalloproteinases-3 that is mutated in Sorsby fundus dystrophy, an autosomal dominant retinopathy of late onset associated with macular degenerative disease [15]. *VMD2* and *TIMP3* SNPs were genotyped in the primary EMSCOT cohort only. To avoid extensive amounts of multiple testing in examining the specific hypothesis that *ABCA4* and *COL2A1* are candidate susceptibility genes for congenital toxoplasmosis we did not look at other eye disorder genes. Table S1 provides details on bp location in the genome (NCBI Build 36.2), position within the gene/locus, alternative alleles and their frequencies (EMSCOT, NCCCTS and public domain) at each SNP. Figure 1 provides a diagrammatic representation of the position of each SNP across the *ABCA4* and *COL2A1* genes, as well as linkage disequilibrium between markers determined and graphed using Haploview available from the HapMap Project Site (http://www.hapmap.org/). All SNPs were genotyped using TaqMan® SNP Genotyping Assays or Custom TaqMan® SNP Genotyping Assays (*COL2A1* rs1635544 only) (Applied Biosystems, CA, USA). Taqman assays were performed in 384-well plates with all liquid handling carried out using a BiomekFX robotics system (Beckman, High Wycombe, UK). Taqman assays were analysed using an ABI 7900HT Fast Real-Time PCR System (Applied Biosystems) and data scored for genotype clustering using ABI SDS v2.1 software.

### Statistical Analyses

Tests for deviation from Hardy-Weinberg equilibrium (HWE) for all markers were performed within STATA v8.0 (http://www.stata.com/) using the GenAssoc package (available from http://www-gene.cimr.cam.ac.uk/clayton/software/stata/) or using routines available from the population genetics R library version 1.2.0 developed by Gregory Warnes and Friedrich Leisch available from http://cran.r-project.org/src/contrib/Descriptions/genetics.html. All markers were in HWE for genetically independent individuals within country-specific samples for EMSCOT, and for the parents of trios in the NCCCTS cohort (data not shown). Linkage disequilibrium was measured as D' [16] and plots were generated using Haploview available from the HapMap Project Site (http://www.hapmap.org/). Power calculations for case-control samples were performed in excel using a script prepared in-house at CIMR by Dr Heather Cordell. Full details are provided in the Online Supplementary Material (Tables S2A to S2D). As examples from these tables, for an effect size (odds ratio or OR) of 3 (i.e. of the order of magnitude of observed OR) at allele frequencies 0.2 and *P* = 0.001, the European study sample had 100% power to detect allelic association for the comparison of uninfected mothers or children (N = 225) against infected mothers or children (N = 232); 98% power for the comparison of unaffected mothers/children (N = 153) against affected mothers/children (N = 79); 91% power for the comparison of unaffected mothers/children (N = 153) against eye lesion mothers/children (N = 53); and 87% power for the comparison of unaffected mothers/children (N = 153) against brain lesion mothers/children (N = 45). TDT power approximations for trios were carried out using the method of Knapp [17] (see Table S3). For example, the 124 affected (i.e. eye and or brain lesions), 113 eye lesion, and 103 brain lesion full child/parent trios had 81.5%, 76.3% and 68% power respectively to detect allelic association at an odds ratio of 3 at *P* = 0.001 for markers at minor allele frequencies = 0.2. All except two exonic SNPs in COL2A1 (rs2070739 both cohorts; rs3803183 Europe only) had variant allele frequencies ≥ 0.2 in both European and North American populations we studied (Table S1).

For the European study sample, genotype and allele frequencies were compared using logistic regression analysis under a multiplicative model (i.e. two alleles contribute twice the effect of one allele), with test statistic (Chi-squared, *χ^2^*), odds ratio (OR), 95% confidence intervals (CI) and *P* values used to evaluate significance of an association. A likelihood ratio test was used to determine dominance effects by comparing 1 degree of freedom (df; allele-wise) and 2 df (genotype-wise) tests. For single marker analysis, logistic regression analyses were carried out with and without adjustment for country, trimester of pregnancy at seroconversion of the mother, and country of origin of grandparents. Since these adjustments had only minor effects on the associations and significance levels obtained without adjustment (i.e. we never observed significance in the unadjusted data that was not also present after adjustment; data not shown), stepwise and interaction analyses were performed without adjusting for these parameters. A stepwise logistic-regression procedure was used to evaluate the relative importance of variants within and between the two candidate gene loci. Wald *χ* ^2^ tests were used to compare models in which the main effects for both loci were modeled with one in which the main effects at the primary locus only were included. Interaction between loci was also analysed using logistic regression analysis.

For the North American study sample, family-based allelic association tests based on the TDT but generalised to allow analysis under additive and dominant models of inheritance were performed within FBAT [18]. Allelic associations, and relative risk estimates, were obtained by creating a case/pseudocontrol study where the cases comprise the genotypes of the affected offspring, and the pseudo-sib controls are the one to three other genotypes (depending on whether phase is known or inferred) which the affected offspring might have received from the parents. The relative risks were estimated using conditional logistic regression analysis, with test statistic (*χ* ^2^), OR, 95% CI and *P* values used to evaluate significance of an association. A stepwise conditional logistic-regression procedure was used to evaluate the relative importance of variants within and between candidate gene loci, as before. Case/pseudocontrol statistical tests implemented within STATA were developed by Heather Cordell and David Clayton at the Cambridge Institute for Medical Research and are available at http://www-gene.cimr.cam.ac.uk/clayton/software/.

Since there was *a priori* clinical evidence to support candidacy of each of the genes studied, and not all markers within genes were independent (i.e. they were in linkage disequilibrium; Figure 1), we did not apply a multiple testing correction. However, the stepwise logistic regression analyses permitted us to determine whether markers within each gene showed independent main effects.

To obtain statistical evidence for imprinting, we used a log-linear method designed to evaluate parent-of-origin effects in case-parent trios [19] once mother's and child's genotypes have been included in the model. We used the program LEM [20] which also allows assessment of maternal genotype effects with or without including a parent-of-origin effect. For the European cohort, where only mother-child pairs were available, the same parameterization was employed and the models were fitted using an in-house program written by Dr Heather Cordell, under the assumption of random mating and Hardy Weinberg equilibrium using public domain allele frequencies for European populations (Table S1).

### Experimental Evidence for Imprinting

Anonymised EBV, Y79 human retinoblastoma (ECACC), and WERI-RB1 human retinoblastoma (gift from Prof D. Trump, Manchester) cell lines were cultured in RPMI 1640-Dutch modification media. HEK293 human embryonic kidney cells (gift from Dr C. Vacher, Cambridge) were cultured in DMEM media. All cell cultures were supplemented with 10% FCS, 100 U/ml Penicillin/100 µg/ml Streptomycin and 2 mM L-glutamine. All reagents were obtained from Invitrogen. DNA was extracted from cell pellets using the salting out method. Total RNA was extracted using TRI-REAGENT (Sigma) according to manufacturer's instructions. Genomic DNA and cDNA from H9 and hSF6 human embryonic stem cells (gift from Prof R. Pederson, Cambridge) were obtained as previously described [21]. Tissue RNAs (placenta, uterus, adult and fetal brain), were part of a total human RNA master panel II (BD Biosciences). To look for monoallelic expression, total RNA (1–2 µg) was reverse transcribed using M-MLV first strand cDNA synthesis system (Invitrogen) using either oligo dT_(18)_ or gene specific primers for *ABCA4* or *COL2A1* (Table S4A). Resulting cDNAs and genomic DNAs were amplified across regions containing polymorphic exonic SNPs of interest using primers as detailed (Table S4B). All PCRs were performed using a Touchdown program, briefly annealing temperature is ramped down by 0.5°C per cycle from 63°C to 56°C followed by a further 19 cycles at 56°C. PCR products were purified using SAP (1 U/µl)//Exo I (10 U/µl) digestion and sequencing performed in both forward and reverse directions using BigDye Terminator v3.1 (Applied Biosystems) and run on an ABI 3100 Genetic Analyser. Sequence data was analysed using pregap4 and gap4.

## Results

### Characteristics of the Two Cohorts Studied

DNA was successfully obtained from 457 mother-child pairs with confirmed infection during pregnancy from the EMSCOT cohort [1], [2]. Transmission of infection during pregnancy was confirmed in 232 (51%) infants; 225 (49%) infants remained uninfected. All infants were monitored for clinical signs until at least three years of age, and many have been followed for >10 years. Of the 232 infected infants, 79 (34%) had clinical signs (referred to as affected) of congenital toxoplasmosis: 53 (67%) with ocular lesions (retinochoroidal lesions), 45 (57%) with brain lesions (hydrocephalus or intracranial calcifications detected on ultrasound examination of the brain), 19 (24%) of these infants had both eye and brain lesions. The median age at the first detection of retinochoroidal lesions was 84 months (range 0 to 237 months; inter-quartile range 46 to 144 months) and at the last negative ophthalmic examination in unaffected children was 31months (range 0 to 252; inter-quartile range 12 to 66 months). 153 (66%) infected infants had no clinical signs of disease up to a minimum follow-up period of 3 years of age. Two-thirds of mother-child pairs reported grandparents' countries of birth. Of these, 95% were Caucasian and 5% of African origin.

For the second independently ascertained cohort, the NCCCTS [3], DNA was successfully obtained from 149 children with confirmed congenital infection (69% Caucasian, 15% Hispanic, 8% Asian or Pacific Islander, 3% African American, 0.7% Native American, 4.7% mixed race) plus available parents. At birth or time of diagnosis, 92 (62%) infected children had brain calcifications with/without hydrocephalus and retinal lesions, 21 (14%) had retinal lesions only, 11 (7%) had brain calcifications with/without hydrocephalus only, and 25 (17%) infected children were without these clinical findings. Only the 124 children with confirmed clinical findings in eye and/or brain were included in the allelic association analysis for this cohort. This provided data on 124 trios classified as affected (i.e. eye or brain lesions or both), 113 trios classified as eye disease (with or without brain disease) and 103 trios classified as brain disease (with or without eye disease).

### Genetic Associations Between ABCA4 or COL2A1 and Eye or Brain Findings in Children with Congenital Toxoplasmosis

For the EMSCOT cohort, no significant associations were observed for any SNPs at *ABCA4* or *COL2A1* when congenitally infected children of mothers with primary gestational infection were compared with children of mothers with primary gestational infection who did not transmit infection to the fetus (Table 1 and data not shown). This was as expected since it was unlikely that genes normally associated with genetic eye disorders would influence transmission of infection from mother to child. Nominally significant (i.e. *P* values without correction for multiple testing) allelic associations were observed for 2 SNPs at *ABCA4* (rs2997633, rs3112831) and 1 SNP at *COL2A1* (rs2276455) when affected children (i.e. children with retinal or brain disease or both) in the EMSCOT cohort were compared with infected but unaffected children (Table 1). Large effect sizes were observed for these associations, particularly in children homozygous for the disease allele (Table 2, odds ratios 6.81, 2.86 and 3.63 respectively for rs2997633, rs3112831 and rs2276455). *ABCA4* rs2297633 retained significance, and an additional SNP at each marker (*ABCA4* rs1761375 and *COL2A1* rs1793958) was found to be associated with disease, when the analysis was enriched for children with the eye lesion phenotype (with/without brain lesions, i.e. leaving out children with brain disease only) compared to unaffected children (Table 1). No associations were observed when the analysis was similarly enriched for children with brain lesions (with/without eye lesions, leaving out children with eye disease only) compared to unaffected children. No associations for any phenotype were observed for *VMD2* or *TIMP3* (data not shown), which are genes associated with other ocular phenotypes that would not have been predicted to play a role in ocular disease associated with congenital toxoplasmosis. All analyses for the EMSCOT study were adjusted for country of birth, trimester of seroconversion, and country of origin of grandparents as a surrogate for ethnicity.

**Table 1:** Logistic regression analysis to look for effect of childs' alleles at *ABCA4* and *COL2A1* on clinical outcome in children in the EMSCOT cohort who became infected *in utero*.

Gene/SNP	Associated Allele^a^	Odds Ratio	95% CI	*P* value	Phenotype^b^
***ABCA4*** **/rs2297633**	-	-	-	**-**	**Infected**
	G	1.96	1.67–3.28	**0.011**	**Affected**
	-	-	-	**-**	**Brain**
	G	2.06	1.14–3.73	**0.017**	**Eye**
***ABCA4*** **/rs1761375**	-	-	-	**-**	**Infected**
	-	-	-	**-**	**Affected**
	-	-	-	**-**	**Brain**
	G	1.73	1.00–3.00	**0.049**	**Eye**
***ABCA4*** **/rs3112831**	-	-	-	**-**	**Infected**
	C	1.58	1.01–2.48	**0.046**	**Affected**
	-	-	-	**-**	**Brain**
	C	1.55	0.93–2.58	**0.094**	**Eye**
***ABCA4*** **/rs952499**	-	-	-	**-**	**Infected**
	T	1.48	0.98–2.25	**0.062**	**Affected**
	-	-	-	**-**	**Brain**
	-	-	-	**-**	**Eye**
***COL2A1*** **/rs2276455**	-	-	-	**-**	**Infected**
	G	1.69	1.07–2.66	**0.024**	**Affected**
	-	-	-	**-**	**Brain**
	G	1.61	0.97–2.68	**0.065**	**Eye**
***COL2A1*** **/rs1793958**	-	-	-	**-**	**Infected**
	-	-	-	**-**	**Affected**
	-	-	-	**-**	**Brain**
	**G**	**1.79**	**1.06–3.04**	**0.031**	**Eye**

SNP = single nucleotide polymorphism; OR = odds ratio; CI = 95% confidence intervals; *P* = probability. Data shown only markers were *P*<0.1 was obtained for analysis by childs' alleles or mothers' alleles (see Table 4), *P*≤0.05 is shown in bold. No associations were observed at *VMD2* or *TIMP3* for any phenotype (data not shown). All analyses for the EMSCOT study were adjusted for country of birth, trimester of seroconversion, and country of origin of grandparents as a surrogate for ethnicity.

a Details of allele frequencies provided in Table S1.

b Phenotypes of children infected *in utero* from mothers infected during pregnancy. Table S8 provides full details of the numbers of children carrying each genotype for each phenotypic group.

**Table 2:** Logistic regression analysis for genotype-wise associations between *ABCA4* and *COL2A1* and risk of congenital toxoplasmosis in the EMSCOT mother-child pairs.

SNP Database Identity	Genotype	OR Disease Child			OR Protection Child			OR Disease Mother			OR Protection Mother		
		OR	95%CI	*P*	OR	95%CI	*P*	OR	95%CI	*P*	OR	95%CI	*P*
*ABCA4*.rs2297633	GG	**6.81**	**1.46–31.7**	**0.0145**				**8.84**	**1.88–41.6**	**0.0058**			
	GT	**4.74**	**0.98–23.0**	**0.0534**	0.70	0.35**–**1.40	0.3102	3.19	0.65**–**15.8	0.1542	**0.36**	**0.17–0.76**	**0.0072**
	TT				**0.15**	**0.03–0.68**	**0.0145**				**0.11**	**0.02–0.53**	**0.0058**
*ABCA4*.rs1761375	GG	1.98	0.69**–**5.61	0.1998				**8.92**	**1.94–41.1**	**0.0050**			
	GA	1.43	0.48**–**4.28	0.5252	0.72	0.36**–**1.44	0.3528	3.03	0.63**–**14.6	0.1660	**0.34**	**0.17–0.69**	**0.0026**
	AA				0.50	0.18**–**1.43	0.1998				**0.11**	**0.02–0.52**	**0.0050**
*ABCA4*.rs3112831	CC	**2.86**	**1.02–7.93**	**0.0439**				**3.12**	**1.01–9.60**	**0.0472**			
	CT	2.13	0.73**–**6.22	0.1670	0.75	0.37**–**1.49	0.4054	1.62	0.51**–**5.21	0.4165	0.51	0.26**–**1.03	0.0615
	TT				**0.35**	**0.13–0.97**	**0.0439**				**0.32**	**0.10–0.98**	**0.0472**
*ABCA4*.rs952499^a^	TT	2.20	0.96**–**5.09	0.0634				**3.66**	**1.50–8.89**	**0.0042**			
	TC	1.50	0.67**–**3.37	0.3203	0.68	0.33**–**1.40	0.2967	1.61	0.71**–**3.61	0.2526	**0.44**	**0.20–0.94**	**0.0345**
	CC				0.45	0.20**–**1.05	0.0634				**0.27**	**0.11–0.66**	**0.0042**
*COL2A1*.rs2276455	GG	**3.63**	**1.29–10.21**	**0.0144**				**3.98**	**1.45–10.97**	**0.0074**			
	GA	**3.13**	**1.12–8.77**	**0.0292**	0.86	0.42**–**1.74	0.6839	1.67	0.63**–**4.41	0.3038	**0.42**	**0.19–0.94**	**0.0342**
	AA				**0.27**	**0.09–0.77**	**0.0144**				**0.25**	**0.09–0.69**	**0.0074**

Results are for genotype associations comparing affected children against unaffected children from infected mothers, and mothers of affected children against mothers of unaffected children. OR = odds ratio; CI = confidence intervals; *P* = probability. Bold indicates significant at *P*≤0.05.

a Significant genotype-wise associations observed for mothers of children with brain lesions but no eye lesions followed the same pattern with significant protection in heterozygous mothers of affected children (data not shown). All analyses were adjusted for country of birth, trimester of seroconversion, and country of origin of grandparents as a surrogate for ethnicity. Table S8 provides full details of the numbers of children and mothers (respectively) carrying each genotype for each phenotypic group.

In the NCCCTS cohort, significant allelic associations were observed for 1 SNP at *ABCA4* (rs952499) and 5 SNPs at *COL2A1* (rs6823, rs2070739, rs2276454, rs1635544, rs3803183) under a dominant model of inheritance (Table 3). The use of case/parent trios and transmission disequilibrium testing (in FBAT) controlled for ethnic admixture in this cohort. For *COL2A1*, SNPs rs2276455, rs2276454 and rs1635544 significance improved when the analysis was enriched for the eye lesion phenotype (with/without brain lesions), consistent with the strong haplotype block formed by these 3 markers (Figure 1). Some significance was also retained in the allelic association analysis (Table 3) when the analysis was enriched for the brain lesion phenotype (with/without eye lesions), which could reflect the larger proportion of children with more severe disease involving both eye and brain lesions in this cohort. There were insufficient children with brain disease only to carry out a separate analysis.

**Table 3:** FBAT analysis under dominant model of inheritance for associations between *ABCA4* and *COL2A1* and congenital toxoplasmosis in the NCCCTS child-parent trios.

Gene/SNP	Allele^a^	Z score	*P* value	Phenotype
*ABCA4*/rs952499	C	−2.255	0.024	Affected^b^
	C	−1.750	0.080	Brain
	C	−1.750	0.080	Eye
*COL2A1*/rs6823	G	+1.949	0.051	Affected
	G	+2.236	0.025	Brain
	G	+1.706	0.088	Eye
*COL2A1*/rs2070739	T	+2.556	0.011	Affected
	T	+2.224	0.026	Brain
	T	+2.224	0.026	Eye
*COL2A1*/rs2276455	-	-	-	Affected
	-	-	-	Brain
	A	−2.213	0.027	Eye
*COL2A1*/rs2276454	A	−2.682	0.007	Affected
	A	−2.813	0.005	Brain
	A	−2.964	0.003	Eye^c^
*COL2A1*/rs1635544	C	−2.269	0.023	Affected
	C	−2.236	0.025	Brain
	C	−2.683	0.007	Eye^c^
*COL2A1*/rs3803183	T	+2.449	0.014	Affected
	T	+2.524	0.012	Brain
	T	+2.226	0.026	Eye

A positive Z score indicates association with disease; a negative Z score indicates the non-associated allele; only Z scores for *P*<0.1 are shown, *P*≤0.05 is shown in bold. Significant associations in the NCCCTS cohort for *COL2A1* with infants with brain lesions likely reflect the larger proportion of children with both brain and eye lesions. There were too few children with brain only or eye only lesions to analyze these as separate groups. The use of trios in the NCCCTS cohort was robust to ethnic admixture.

a Details of allele frequencies provided in Table S1.

b Only significant under dominant model, disease allele recessive; using case/pseudo-control analysis, OR for heterozygote compared to homozygous disease allele is 0.37 (95% confidence intervals: 0.14–0.92; *P* = 0.032).

c Also significant under additive model; OR for carrying disease allele is 2.45 (1.22–4.94; *P* = 0.012) for rs2276454 and 2.6 (1.25–5.39; *P* = 0.010) for rs1635544. Table S9 provides full details of the genotypes of child/parent trios, including phenotype of the child.

Overall, the evidence from the children with eye and brain signs associated with congenitally acquired toxoplasmosis in these two cohorts was for association with eye disease and these two previously recognized eye disorder genes, *ABCA4* and *COL2A1*. Linkage disequilibrium patterns across both loci were similar in the two cohorts, with all of the SNPs that contribute main effects (as determined by stepwise logistic regression analysis; legend to figure 1 and Online Supplementary Material, Text S1 and Tables S5,S6,S7) in the associations in each cohort falling in the same haplotype blocks (Figure 1) within these large genes: *ABCA4* spanning 128.3 kb with 50 exons, 2,273 amino acids, 255.9 kDa protein; *COL2A1* spanning 31.5 kb with 54 exons, 1,418 amino acids, 134.4 kDa protein. Inter-locus stepwise logistic regression analysis showed that the two genes had independent effects (Tables S5 and S6), and there was no statistical evidence for interaction between them (data not shown).

### Influence of Mother's Genotype on Clinical Outcome in Children with Congenital Toxoplasmosis

Additional genetic information available in the EMSCOT cohort was that of mother's genotype. As a first approach to determining whether mother's genotype had an effect on disease outcome in the child, logistic regression analysis was carried out comparing mothers of affected children (eye and/or brain disease) with mothers of infected unaffected children. This proved interesting in that (1) all of the allele-wise associations were more significant in comparisons of mothers (Table 4) than in comparisons of infants (Table 1), (2) additional *ABCA4* (rs952499) and *COL2A1* (rs2070739, rs1635544) were associated with mothers of affected infants compared to mothers of unaffected infants (Table 4), and (3) all of the *ABCA4* SNPs but none of the *COL2A1* SNPs were associated with mothers of infants with brain disease (Table 4, note also footnote on mothers of children with brain disease only). Statistically, it appeared that genetic effects of these two loci on disease in the child were being diluted out in making direct comparisons of the affected versus unaffected children relative to the evidence for association when comparing the mothers. Unusual patterns of inheritance were also observed when comparing genotype-wise associations in children versus mothers (Table 2) suggesting that there may be a direct effect of mother's genotype, or that there may be parent-of-origin effects (imprinting), i.e. that for the child it is the origin of, and not just the combination of, alleles that is important in determining disease risk. Using a log-linear method previously designed to evaluate maternal genotype and/or parent-of-origin effects in case-parent trios [19], evidence for imprinting given mother's and child's genotypes was obtained at *ABCA4* rs952499 (*P* = 0.033) and *COL2A1* rs2070739 (*P* = 0.05) in the NCCCTS trios. For EMSCOT we adapted the method for use with mother-child pairs and found evidence for effects of maternal genotype given child's genotype and imprinting for both *COL2A1* rs1635544 (*P* = 0.0088) and rs3803183 (*P* = 6.87×10^−5^), and evidence for imprinting taking account of both mother's and child's genotype for *COL2A1* rs3803183 (*P* = 0.0025). Given the statistical limitations of small sample size and power in the modeling analysis, and the fact that a direct effect of mother's genotype seemed unlikely biologically, we looked for experimental evidence of epigenetic effects or imprinting for *ABCA4* and *COL2A1*.

**Table 4:** Logistic regression analysis to look for effect of mothers' alleles at *ABCA4* and *COL2A1* on clinical outcome in children in the EMSCOT cohort who became infected *in utero*.

Gene/SNP	Associated Allele^a^	Odds Ratio	95% CI	*P* value	Phenotype
*ABCA4*/rs2297633	-	-	-	-	Infected
	G	2.87	1.61–5.09	0.0003	Affected
	G	2.54	1.23–5.28	0.012	Brain
	G	3.09	1.56–6.16	0.001	Eye
*ABCA4*/rs1761375 c	-	-	-	-	Infected
	G	2.96	1.70–5.17	0.0001	Affected
	G	3.95	1.80–8.67	0.0006	Brain
	G	2.50	1.34–4.660	0.004	Eye
*ABCA4*/rs3112831	-	-	-	-	Infected
	C	1.82	1.12–2.97	0.015	Affected
	C	2.05	1.08–3.86	0.027	Brain
	C	1.86	1.06–3.27	0.029	Eye
*ABCA4*/rs952499 c	-	-	-	-	Infected
	T	1.93	1.23–3.02	0.004	Affected
	T	2.24	1.26–3.99	0.006	Brain
	T	1.60	0.98–2.63	0.061	Eye
*COL2A1*/rs2070739	-	-	-	-	Infected
	T	2.06	1.0–4.28	0.051	Affected
	-	-	-	-	Brain
	-	2.92	1.34–6.34	0.007	Eye
*COL2A1*/rs2276455	-	-	-	-	Infected
	G	2.05	1.24–3.39	0.005	Affected
	-	-	-	-	Brain
	G	2.06	1.16–3.66	0.014	Eye
*COL2A1*/rs1635544	-	-	-	-	Infected
	-	-	-	-	Affected
	-	-	-	-	Brain
	T	2.57	1.26–5.26	0.010	Eye
*COL2A1*/rs3803183	-	-	-	-	Infected
	T	2.83	1.42–5.61	0.003	Affected
	-	-	-	-	Brain
	T	2.09	1.04–4.25	0.039	Eye

SNP = single nucleotide polymorphism; OR = odds ratio; CI = 95% confidence intervals; *P* = probability. Data shown only for markers were *P*<0.1 was obtained for analysis by childs' alleles (see Table 1) or mothers' alleles, *P*≤0.05 is shown in bold. All analyses for the EMSCOT study were adjusted for country of birth, trimester of seroconversion, and country of origin of grandparents as a surrogate for ethnicity.

a Details of allele frequencies provided in Table S1.

b Phenotypes of children infected *in utero* from mothers infected during pregnancy.

c Significant allele-wise associations were also observed at *ABCA4* rs1761375 and rs952499 for mothers of children with brain lesions but not eye lesions (data not shown). None of the *COL2A1* associations in EMSCOT were significant when the analysis was stratified for mothers of children with brain lesions (data not shown). Table S8 provides full details of the numbers of mothers carrying each genotype for each phenotypic group.

### Experimental evidence for imprinting

**Figure 2:**
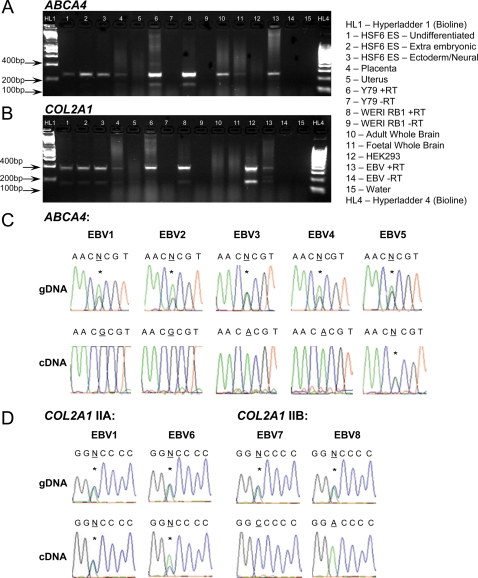
Experimental evidence for monoallelic expression at *ABCA4* and *COL2A1*. (A) and (B) show transcripts of *ABCA4* and *COL2A1*expressed in human embryonic stem cell lines (HSF6 shown here; H9, data not shown) before (lane 1) and after commitment to extra-embryonic (lane 2) or ectoderm/neural (lane 3) lineages, in human placenta (lane 4) but not uterus (lane 5). *ABCA4* with exon 10 is expressed in Y79 (lane 6) and WERI RB1 (lane 8) eye cell lines, adult (lane 10) and fetal (lane 11) brain, and EBV lines (e.g. lane 13) used for sequencing (C), but not in HEK293 (lane 12). The isoform without exon 10 is seen in eye lines (lanes 6, 8). *COL2A1 i*soform IIA is expressed in Y79 (lane 6) and WERI RB1 (lane 8) cells. Neither isoform is expressed in adult (lane 10) or fetal (lane 11) brain. Both are expressed in HEK293 (lane 12), and in EBV lines (e.g. lane 13) used for sequencing (D). Water and –RT lanes are indicated. (C) and (D) show sequence analysis of genomic DNA (gDNA) and cDNA in EBV lines heterozygous for exonic SNPs. (C) EBV lines heterozygous for *ABCA4* rs3112831 in gDNA; lines EBV1 to EBV4 homozygous (i.e. monoallelic) in cDNA specific for the exon 10-containing isoform. Line EBV5 is heterozygous, indicating that mono-allelic silencing is polymorphic. (D) EBV lines show monoallelic expression for *COL2A1* SNP rs3737548 in PCR products specific for isoform IIB, but not IIA. Positions of SNPs (*) indicated by N where heterozygous, with the bp underlined for mono-allelic expression in cDNA.

To obtain experimental evidence for imprinting, we screened an anonymised EBV B cell bank (a) to find individuals heterozygous for exonic SNPs at both loci; and (b) to determine mono-allelic expression in elicit transcripts obtained from RNA from these EBV cell lines. EBV lines were identified that were heterozygous for SNP rs3112831 in exon 10 of *ABCA4* and for SNP rs3737548 in exon 7 of *COL2A1*. RT/PCR analysis demonstrated that *ABCA4* and *COL2A1* were expressed in human embryonic stem cell lines before and after commitment to extra-embryonic or ectoderm/ neural lineages, and in human placenta but not uterus (Figure 2A and 2B). Two isoforms, with or without exon 10, were observed for *ABCA4*. The exon 10-containing isoform was strongly expressed in Y79 and WERI RB1 eye cell lines. This isoform was also expressed in adult and fetal brain, and in EBV cell lines used for sequence analysis, but not in HEK293 cells. The isoform without exon 10 has not been previously reported. Since there is no expressed sequence tag cDNA or RNA evidence for this spliced variant reported in the ENSEMBL genome database, we cannot be certain that this transcript is translated into a functional protein. For the known functional exon 10-containing isoform at *ABCA4* we found that 4 of the 5 EBV lines that were heterozygous for genomic DNA showed monoallelic expression in cDNA for the exon 10 rs3112831 SNP (Figure 2C). This mono-allelic expression was observed independently in multiple RNA extractions from each of which multiple cDNA preparations were made from these EBV cell lines, demonstrating that this was not due to chance events in amplification of the cDNA. Examining parental genotypes in the EBV cell bank, we determined that the paternally-derived allele is silenced for the exon10-containing isoform of *ABCA4*. However, given the small sample of polyclonal EBV cell lines examined we cannot state definitively that it is always the paternally-derived allele that is silenced. Hence, this could represent random choice autosomal monoallelic expression, which has recently been shown to be more common in the genome than was previously supposed [22]. This could also account for the apparent polymorphic nature of the silencing, since a majority of genes showing random choice autosomal monoallelic expression display biallelic expression in some clonal cell lines. Since all the EBV cell lines employed here were polyclonal, imprinting currently provides the more likely explanation for the monoallelic expression we observed.

We also demonstrated (Figure 2D) monoallelic expression for a SNP in exon 7 in cDNA specifically amplified for the IIB short form of *COL2A1*, but not for cDNA amplified for the IIA long form, consistent with isoform-specific imprinting in this case with the maternally-derived allele silenced. This fits with the observation that *COL2A1* lies within a cluster of genes on human chromosome 12q13.11 syntenic with mouse chromosome 15 band F2 that are known to be maternally imprinted [23], although again we cannot definitively exclude autosomal random mono-allelic expression.

## Discussion

Herein we examined the specific genetic hypothesis that polymorphisms in two genes known to be associated with ocular disease, *ABCA4* and *COL2A1*, are associated with ocular disease caused by congenital toxoplasmosis. Evidence for genetic associations observed initially in a European cohort was replicated in an independently ascertained cohort from North America. One value of genetic association studies is that they provide concrete insight into processes that determine clinical outcome of disease, in this case events that may occur in embryonic development when it is not easy to determine what is happening when the fetus is infected with a parasite such as *T. gondii*, or what the parasite is doing during early post-natal development. We chose to look specifically for associations with *ABCA4* and *COL2A1* (a) because both had defined single gene disorders that result in congenital or early onset ocular disease; and (b) some parallels in clinical pathology or putative pathogenic mechanisms could be drawn between the genetic disorders and ocular disease induced by *T. gondii* infection *in utero*. Although broadly defined as retinochoroiditis, ocular disease caused by toxoplasmosis is associated with a wide range [24]–[28] of ophthalmological vitreoretinal pathologies including retinal necrosis with adjacent choroiditis, and less frequently, vasculitis, hemorrhage, choroidal neovascularization, vitritis, posterior vitreous detachment, thinning of the retina, retinal detachment, optic nerve changes, cataracts, glaucoma and myopia. Ocular features of Stickler's syndrome and other *COL2A1* genetic disorders (reviewed [29]) commonly include myopia, vitreoretinal degeneration, retinal thinning, retinal detachment, cataract, and glaucoma. Genetic disorders of *ABCA4* are associated with juvenile onset retinal dystrophies including Stargardt's disease. Population frequencies of known coding region mutations causing the clinical genetic disorders of Stargadt's disease or Stickler's syndrome are not sufficient to account for the association with clinical outcome of *T. gondii* infection we observe here. More likely, the genetic associations that we have observed will reflect subtle regulatory polymorphisms that lie outside the coding region and influence gene expression and possibly imprinting. The benefit of finding a genetic association with specific genes is that we can begin to understand more about how the parasite triggers the development of ocular disease during embryogenesis and fetal development by examining what is known about the chronological expression and localization of COL2A1 and ABCA4 during development and after birth.

*COL2A1* encodes type II collagen which is found in the vitreous humor, cornea, sclera, lens, ciliary body, retinal pigment epithelium, and retina of the eye [29]. During embryogenesis, type IIA procollagen is localized around cells of the developing ganglion cell layer [30], playing a role in guiding the axonal processes of the developing retinal ganglion cells as they traverse the retinal surface and converge to form the optic nerve bundle [31]. Both IIA and IIB transcripts occur in retinal pigment epithelial cells, with the protein localized around these cells during embryogenesis, acting to maintain the structural strength of the attachment area of retina and pigment epitheliuim [32], [33]. In the mouse eye, transcripts for types IIA and IIB mRNA have been detected during embryogenesis starting at least from day 10.5 (equivalent to Carnegie Stage 11; ∼23–26 days in human embryogenesis), with the relative levels of both isoforms remaining fairly constant during normal embryonic development, and with the type IIB isoform slightly predominating [33]. The critical period for the development of major congenital eye disorders is during weeks 4 to 8 of gestation (http://www.bioscience.org/atlases/fert/embrper.htm). The genetic association between ocular disease in congenital toxoplasmosis and *COL2A1* might reflect differences in collagen expression in the retina and vitreous influencing migration or dissemination of the parasite within the eye, or stability of the eye structures when there are multiplying parasites in the choroid, retina or optic nerve. This would be likely to have its most profound effect on pathology during early embryogenesis when the eye is forming and COL2A1 is first expressed. Since toxoplasmosis is a complex disease, multiple genes and modifiers will contribute to end stage pathology. Hence, we do not expect that end stage pathology seen in congenital toxoplasmosis will be the same as that observed in the case of a very specific genetic disorder like Stickler's disease. Nevertheless, differences in collagen expression could contribute to similar end stage consequences of disease (e.g. retinal detachment) that occur rarely in toxoplasmosis (<10% of those with the most severe congenital toxoplasmosis) but commonly (>70%) in Sticklers disease, despite very different morphology of the vitreous and retinal findings, e.g. discrete chorioretinal lesions and optic neuritis and inflammatory cells in the vitreous for toxoplasmosis and lattice retinal degeneration with bands in vitreous for Stickler's disease. Differences in collagen expression might also affect angiogenesis and fibrogenesis, which both appear to affect ophthalmologic sequellae [34]. It is of interest in this regard that host collagen genes are among those with increased expression when *T. gondii* infects fibroblasts [35].

Levels of type II collagen mRNA decline in the eye post-natally, and continue to exhibit a slow age-dependent reduction [32], [33]. However, reactivation of type IIA collagen can occur at later stages, possibly during tissue repair [30], and may also modulate proliferative processes in the vitreous [36]. Genetic differences in COL2A1 type IIA expression, triggered directly by the parasite and/or the tissue response to the parasite, could play a role in the continuing postnatal development of pathology associated with congenitally-acquired toxoplasmosis. Type IIA is the predominant isoform in non-cartilaginous and non-ocular tissues during embryogenesis, but there is a switch from type IIA to IIB as chondrocytes differentiate, making the shorter form IIB the predominant form in mature cartilage. This has important implications in relation to the lack of skeletal features in clinical phenotypes observed in congenital toxoplasmosis, as discussed below in relation to the epigenetic silencing of the maternally-derived allele that we have observed for the type IIB isoform of *COL2A1*.

*ABCA4* encodes a retina-specific ATP-binding cassette transporter protein that is located at the rim of the photoreceptor outer segment disc and is involved in retinoid (*N*-retinylidene-phosphatidylethanolamine) transport across the disc membrane [37]. Less is known about the chronological pattern of expression of ABCA4 in the eye during development compared to COL2A1, although a high frequency of cDNA clones positive for ABCA4 has been reported in screens of cDNA libraries prepared from developing mouse eye [38]. Of interest too is the observation is that ABCA4 is selectively expressed in the choroid plexus throughout development [39], [40], suggesting a possible role for *ABCA4* in determining pathology in brain in addition to eye, that would be consistent with the association observed with hydrocephalus, in particular when examining associations between mother's genotype and clinical outcome in the child for the European cohort.

The unusual patterns of inheritance of disease alleles that we observed when comparing association in the infants with outcomes in infants born to heterozygous mothers led us to consider whether epigenetic effects, specifically imprinting, could be influencing these genetic associations. We found evidence of isoform-specific monoallelic expression of alleles at both genes, which for *ABCA4* was also polymorphic. Isoform-specific [41], [42] and polymorphic [43] imprinting patterns have also been reported in other genes. At *ABCA4* it was the paternally-derived allele for the normally expressed exon 10-containing isoform that was silenced in the polyclonal EBV cell lines that we examined. Although consistent with imprinting, we could not formally rule out random choice autosomal mono-allelic expression. The patterns of monoallelic expression that we observed in EBV cells may not reflect precisely what occurs in the tissue-specific setting *in vivo*. However, if the data for EBV cell lines does parallel the *in vivo* situation, children homozygous for the disease allele will always have a disease allele expressed in the eye or brain during embryogenesis and post-natally, consistent with the high odds ratios (6.81) for disease (Table 2) in children homozygous at SNP *ABCA4* rs2297633 that contributes independent main effects (see Online Supplementary Material) in the EMSCOT cohort. For heterozygous children, expression of the disease allele will be dependent upon which parent it was derived from. At *COL2A1*, only the maternally-derived allele for isoform IIB was silenced in the polyclonal EBV cell lines examined. Skeletal anomalies are never associated with congenital toxoplasmosis. Possible explanations for the observed patterns of association between *COL2A1* and clinical signs in congenital toxoplasmosis are (a) that the etiological variant only influences expression or function of the non-silenced exon 2-containing IIA long-form allele; or (b) the disease-causing variant is common to both isoforms but does not manifest as skeletal abnormalities due to the silencing of isoform IIB expressed in cartilage. This could also explain why Stickler's disease with ocular but no skeletal involvement is not confined to exon 2 variants [44], [45]. Re-sequencing is in progress to identify the etiological variant(s) in our cohorts.

Further work is required to clarify the mechanisms of epigenetic modifications at both *COL2A1* and *ABCA4*, especially during development. Such research will benefit from further analysis of imprinting patterns in animal models of congenital toxoplasmosis, in addition to human cell lines and clinical samples. A key question too is how the parasite influences genetically-regulated pathogenesis of disease, which could be via polymorphisms in NFκB sites that regulate gene expression and developmental processes. *T. gondii* is a potent trigger for, and direct regulator of, this signaling pathway [46], [47] and its presence could upset programming of expression of these two genes, both of which have NFκB transcription factor binding sites in their promoters (Matinspector [48] and Alibaba [49] software; data not shown), during eye or brain development. It is also possible that the parasite may directly interfere with methylation and/or histone acetylation patterns of host DNA, thereby directly affecting epigenetic regulation of gene expression. Overall, our finding that polymorphisms at *ABCA4* and *COL2A1* are associated with ocular and other manifestations of congenital toxoplasmosis provides novel insight into the molecular pathways that can be affected by congenital infection with this parasite.

## Supporting Information

Text S1 Supplementary Results. This file contains the description of results pertaining to Tables S1,S2,S3,S4,S5,S6,S7,S8,S9.
(0.04 MB DOC)

Table S1 Detailed information on SNPs genotyped in the EMSCOT and NCCCTS cohorts
(0.07 MB DOC)

Table S2 Power calculations for case-control samples.
(0.09 MB DOC)

Table S3 Power calculations for the NCCCTS cohort.
(0.07 MB DOC)

Table S4 Gene specific primers for reverse transcription (A); and PCR primers used to obtain products for sequencing (B).
(0.04 MB DOC)

Table S5 Intra-locus and inter-locus forward stepwise logistic regression analysis for allelic associations comparing mothers of affected children with mothers of unaffected children from the EMSCOT cohort.
(0.07 MB DOC)

Table S6 Intra-locus and inter-locus forward stepwise conditional logistic regression analysis in the NCCCTS cohort.
(0.07 MB DOC)

Table S7 Summary of haplotype associations across COL2A1 analysed using TRANSMIT for the NCCCTS cohort.
(0.07 MB DOC)

Table S8 Absolute numbers of individuals with each genotype at each marker according clinical to phenotype and appropriate control group for (A) children and (B) mothers in the EMSCOT study.
(0.19 MB DOC)

Table S9 Absolute numbers of individuals with each genotype at each marker according to clinical phenotype for the 124 (113 and 103) possible children included in the genetic study for the NCCCTS study.
(0.09 MB DOC)
