# Supplementary material for: Genetic and Epigenetic Factors at COL2A1 and ABCA4 Influence Clinical Outcome in Congenital Toxoplasmosis
Source: PLoS One. 2008 Jun 4;3(6):e2285. doi: 10.1371/journal.pone.0002285 (PMC2390765; doi:10.1371/journal.pone.0002285)
Supplement: Text S1 — Supplementary Results. This file contains the description of results pertaining to Tables S1,S2,S3,S4,S5,S6,S7,S8,S9. (0.04 MB DOC) [file pone.0002285.s001.doc]

Supporting Online Results for

**Genetic and epigenetic factors at *COL2A1* and *ABCA4* influence clinical outcome in congenital toxoplasmosis**

Sarra E. Jamieson, Lee-Anne de Roubaix, Mario Cortina-Borja, Hooi Kuan Tan, Ernest J. Mui, Heather J. Cordell, Michael J. Kirisits, E. Nancy Miller, Christopher S. Peacock, Aubrey C. Hargrave, Jessica J. Coyne, Kenneth Boyer, Marie-Hélène Bessieres, Wilma Buffolano, Nicole Ferret, Jacqueline Franck, François Kieffer, Paul Meier, Dorota E. Nowakowska, Malgorzata Paul, François Peyron, Babill Stray-Pedersen, Andrea-Romana Prusa, Philippe Thulliez, Martine Wallon, Eskild Petersen, Rima McLeod, Ruth E. Gilbert and Jenefer M. Blackwell^*^

*To whom correspondence should be addressed. Email: jblackwell@ichr.uwa.edu.au

**This file includes**

Description of supplementary results

Additional references

**SUPPLEMENTARY RESULTS**

Table S1 provides details of the allele frequencies for SNPs for the EMSCOT and NCCCTS cohorts, and frequencies accessible in the public domain. Tables S2 and S3 provide power calculations for the EMSCOT and NCCCTS cohorts, respectively. Table S4 gives information on the primers used for the experimental studies that demonstrated mono-allelic expression in EBV cell lines. Tables S5 and S6 are the results of stepwise logistic regression and stepwise conditional logistic regression carried out on the EMSCOT and NCCCTS data. Table S7 provides the results of haplotype analysis for COL2A1 in the NCCCTS cohort. The stepwise logistic regression and haplotype analyses are discussed in more detail below. Table S8 shows the genotype x phenotype data for EMSCOT, and Table S9 provides the genotype data by families for the NCCCTS cohort.

Stepwise logistic regression analyses [1] were undertaken to determine whether the associations with *ABCA4* and *COL2A1* observed in each cohort showed independent main effects. For the EMSCOT cohort, SNPs rs2997633 and rs1761375 at *ABCA4* both add significant main effects when compared to the other two markers (rs3112831 and rs952499) that showed single point associations, but they did not add significant main effects to each other (Table S5). Neither rs3112831 nor rs952499 added significant main effects to either rs2997633 or rs1761375, or in a model where both rs2997633 and rs1761375 were included. This means that all of the association at *ABCA4* in the EMSCOT cohort is accounted for by rs2997633 and rs1761375, implying that a single etiological variant in strong linkage disequilibrium with these two markers may account for the association in Europe. This is consistent with the observation that (main text Fig. 1) all 4 markers lie within a haplotype block that spans intron 6 to intron 19 of the *ABCA4* gene. Of note, the one non-synonymous coding SNP variant rs3112831 at exon 10 did not account for all of the association within this haplotype block and is therefore unlikely to be the etiological variant. For the NCCCTS cohort, a weak single point association was observed under a dominant model for SNP rs952499 but not the other SNPs (main text, Table 3) within this haplotype block, which was similar in both European and North American samples (main text Fig. 1). This could mean that a different etiological variant is involved, or that a common etiological variant is on a different haplotype in the families contributing to this association in the NCCCTS cohort. This is possible given differences in ethnic composition of the two cohorts. Overall the NCCCTS cohort provides weak but supportive evidence for an effect of *ABCA4* on clinical symptoms associated with congenital toxoplasmosis.

At *COL2A1*, SNPs rs2276455, rs1635544 and rs3803183 all add significant main effects when compared to rs2070739 in the EMSCOT cohort, but rs2070739 does not add significant main effects once any one of these individual markers is included in the model (Table S5). SNPs rs2276455, rs1635544 and rs3803183 generally do not add significant main effects to each other, except for rs1635544 to rs3803181. However, once pairs of these markers are taken into the model, the third SNP does not add significant main effects to any of the pair-wise combinations. SNPs rs2276455 and rs1635544 are in strong linkage disequilibrium with each other as defined by both D’, and there is evidence from the D’ statistic that there may be haplotypes that extend to rs3803183 (main text Fig. 1). A single etiological variant could account for the association with *COL2A1* in the EMSCOT cohort. Neither of the SNPs (rs2070739, rs3803183) that result in non-synonymous amino acid substitutions (Table S1) appears to be the primary functional variant.

For the NCCCTS cohort, additional single point associations (rs6823, rs2276454) were observed at *COL2A1* (main text, Table 3) compared to the EMSCOT cohort (main text, Table 1). As for the EMSCOT cohort, markers rs2276455, rs2276454 and rs1635544 lie in a strong linkage disequilibrium block (main text Fig. 1). However, the observation that rs2276454 is associated with clinical signs of disease in NCCCTS and not EMSCOT immediately suggests different haplotypic structures in the two cohorts. For the NCCCTS cohort, rs2276454 and rs1635544 add significant main effects to rs2070739 (Table S6), but rs2070739 does not add main effects to these two SNPs that lie in the strong central linkage disequilibrium block (main text Fig. 1). Interestingly, rs2070739 does add significant main effects to rs6823, and rs6823 adds separate main effects to both rs2070739 and rs2276455. As a SNP causing a non-synonymous amino acid substitution, rs2070739 could contribute functionally to the association. In this cohort the data from the stepwise analysis is more complex, suggesting more than one etiological variant contributing to the disease association. It should be noted, nevertheless, that rs2276455, rs2276454 and rs1635544 did not add significant main effects to rs6823. Hence, there is a possibility that there may be extended disease associated haplotypes across the *COL2A1* gene, consistent with the D’ linkage disequilibrium analysis. The number of case/parent trios contributing to the analysis did not provide sufficient power to consider more complex models in the stepwise logistic regression analysis, and this may also have contributed to lack of power in the one versus two locus comparisons. The possibility that there is more than one etiological disease variant in this cohort is supported by haplotype analysis in TRANSMIT [2]. Whilst there was a common haplotype (2.2.1.2.2.1.2 or C.C.A.A.C.G.A) significantly associated (χ^2^ = 8.29; *P* = 0.004) with protection (i.e. transmitted less times than expected) across all 7 SNP markers, or all combinations thereof except rs2276455 and rs2276454, haplotypes significantly associated with disease (i.e. transmitted more time than expected) were only observed with the subset of SNPs that included rs2276454 and rs1635544, with weak evidence (*P* = 0.074) for an over transmitted haplotype across rs6823 and rs2070739 (Table S7) consistent with the stepwise conditional logistic regression analysis. Overall, the data from the NCCCTS provided strong single point data supporting the hypothesis that polymorphism(s) at *COL2A1* affect clinical symptoms associated with congenital toxoplasmosis, with additional support for the observation that there may be different and/or multiple disease associated variants within and between the two cohorts. Again, this is possible given differences in ethnic composition of the two cohorts.

Inter-locus comparisons in the NCCCTS cohort (Table S6) demonstrated independent effects when the most significant SNP (rs1635544) at *COL2A1* was added to *ABCA4* rs952499, but not in reverse when dominance was taken into account. Since the effects of these two loci were independent in the larger EMSCOT cohort (Table S5), this could also reflect lack of statistical power.

**References**

1. Cordell HJ, Clayton DG (2002) A unified stepwise regression procedure for evaluating the relative effects of polymorphisms within a gene using case/control or family data: application to HLA in type 1 diabetes. Am J Hum Genet 70: 124-141.

2. Clayton D, Jones H (1999) Transmission/disequilibrium tests for extended marker haplotypes. Am J Hum Genet 65: 1161-1169.

3. Knapp M (1999) A note on power approximations for the transmission/disequilibrium test. Am J Hum Genet 64: 1177-1185.
