# Supplementary material for: Genetic and Epigenetic Factors at COL2A1 and ABCA4 Influence Clinical Outcome in Congenital Toxoplasmosis
Source: PLoS One. 2008 Jun 4;3(6):e2285. doi: 10.1371/journal.pone.0002285 (PMC2390765; doi:10.1371/journal.pone.0002285)
Supplement: Table S1 — Detailed information on SNPs genotyped in the EMSCOT and NCCCTS cohorts (0.07 MB DOC) [file pone.0002285.s002.doc]

**Table S1.** Detailed information on SNPs genotyped in the EMSCOT and NCCCTS cohorts

| SNP | Bp Position | Position in Gene | AA change^a^ | Allele 1 (strand) | Allele 2  (strand) | EMSCOT Allele Frequency | | NCCCTS Allele Frequency | | Public Domain Allele Frequency | | Population ID Public Domain Frequencies (source dbSNP) |
| --- | --- | --- | --- | --- | --- | --- | --- | --- | --- | --- | --- | --- |
|  |  |  |  |  |  | Allele  1 | Allele 2 | Allele 1 | Allele 2 | Allele 1 | Allele 2 |  |
| ***ABCA4* (reverse strand Chr 1p22.1 ):** | | | | | | | | | | | | |
| rs1801574 ^e^ | 94248976 | exon 40 | Leu-1894-Leu | G (-) | C (-) | 0.25 | 0.75 | 0.24 | 0.76 | 0.19 | 0.81 | HapMap CEU |
| rs2275033 | 94252625 | intron 38 | **-** | T (+) | C (+) | 0.41 | 0.59 | 0.34 | 0.66 | 0.43 | 0.57 | HapMap CEU |
| rs2297671 | 94268841 | intron 28 | **-** | G (-) | A (-) | 0.53 | 0.47 | 0.58 | 0.42 | 0.66 | 0.34 | Mixed ^b^ |
| rs2297633 | 94283261 | intron 19 | **-** | T (+) | G (+) | 0.32 | 0.68 | 0.28 | 0.72 | 0.32 | 0.68 | HapMap CEU |
| rs1761375 | 94310599 | intron 11 | **-** | G (+) | A (+) | 0.68 | 0.32 | 0.74 | 0.26 | 0.70 | 0.30 | HapMap CEU |
| rs3112831 ^e^ | 94316822 | exon 10 | Arg-423-His | T (-) | C (-) | 0.66 | 0.34 | 0.71 | 0.29 | 0.73 | 0.27 | HapMap CEU |
| rs952499 | 94331013 | intron 6 | **-** | T (-) | C (-) | 0.51 | 0.49 | 0.51 | 0.49 | 0.55 | 0.45 | HapMap CEU |
| ***COL2A1* (reverse strand Chr 12q13.11):** | | | | | | | | | | | | |
| rs6823 | 46648679 | 3'UTR | **-** | G (+) | C (+) | 0.42 | 0.58 | 0.44 | 0.56 | 0.53 | 0.47 | HapMap CEU |
| rs2070739 ^e^ | 46654243 | exon 53 | Ser-1405-Gly | T (-) | C (-) | 0.12 | 0.88 | 0.17 | 0.83 | 0.08 | 0.92 | HapMap CEU |
| rs2276455 | 46662404 | intron 34 | **-** | A (-) | G (-) | 0.43 | 0.57 | 0.41 | 0.59 | 0.39 | 0.61 | ALFRED ^c^ |
| rs2276454 ^e^ | 46662558 | exon 34 | Gly-2245-Gly | G (+) | A (+) | 0.57 | 0.43 | 0.61 | 0.39 | 0.68 | 0.32 | HapMap CEU |
| rs1635544 | 46666289 | intron 23 | **-** | T (-) | C (-) | 0.53 | 0.47 | 0.55 | 0.45 | 0.48 | 0.52 | HapMap CEU |
| rs1793958 | 46678700 | intron 2 | **-** | G (+) | A (+) | 0.60 | 0.40 | 0.55 | 0.45 | 0.69 | 0.31 | HapMap CEU |
| rs3803183 ^e^ | 46684347 | exon 1 | Ser-9-Thr | T (-) | A (-) | 0.18 | 0.82 | 0.25 | 0.75 | 0.08 | 0.92 | AGI_ASP ^d^ |
| ***VMD2* (forward strand Chr 11q12.2):** | | | | | | | | | | | | |
| rs1800007 | 61475963 | Exon 2 | Leu-37-Leu | T (+) | C (+) | 0.68 | 0.32 | **-** | **-** | 0.70 | 0.30 | HapMap CEU |
| rs909268 | 61480279 | Intron 4 | **-** | T (+) | A (+) | 0.26 | 0.74 | **-** | **-** | 0.18 | 0.82 | CEPH |
| ***TIMP3* (forward strand Chr 22q12.3):** | | | | | | | | | | | | |
| rs130559 | 31518536 | Intron 1 | **-** | T (+) | C (+) | 0.21 | 0.79 | **-** | **-** | 0.20 | 0.80 | HapMap CEU |
| rs242085 | 31551030 | Intron 1 | **-** | T (+) | C (+) | 0.46 | 0.54 | **-** | **-** | 0.52 | 0.48 | HapMap CEU |
| rs1427385 | 31574314 | Intron 1 | **-** | T (+) | C (+) | 0.45 | 0.55 | - | - | 0.38 | 0.62 | HapMap CEU |

^a^ where applicable, ^b^ average of African American, Chinese and Japanese, ^c^ Mixed European sample in ALFRED at http://alfred.med.yale.edu/alfred/,

^d^ AGI_ASP is a 50:50 mix of Caucasian and African American DNA samples, the closest public domain data that we could find to resemble ethnicity for EMSCOT (95:5), for which public domain frequencies were used in the parent-of-origin statistical analysis, ^e^ these SNPs were genotyped to search for heterozygotes in the EBV cell bank.
