# Supplementary material for: Genetic and Epigenetic Factors at COL2A1 and ABCA4 Influence Clinical Outcome in Congenital Toxoplasmosis
Source: PLoS One. 2008 Jun 4;3(6):e2285. doi: 10.1371/journal.pone.0002285 (PMC2390765; doi:10.1371/journal.pone.0002285)
Supplement: Table S2 — Power calculations for case-control samples. (0.09 MB DOC) [file pone.0002285.s003.doc]

**Table S2** Power calculations for case-control samples.

| **SNP Allele Freq** | **Effect Size**  **(Odds Ratio)** | **232 Cases vs. 225 Controls** | | | **79 Cases vs. 153 Controls** | | | **53 Cases vs. 153 Controls** | | | **45 Cases vs. 153 Controls** | | |
| --- | --- | --- | --- | --- | --- | --- | --- | --- | --- | --- | --- | --- | --- |
|  |  | ***P* = 0.05** | ***P* = 0.01** | ***P* = 0.001** | ***P* = 0.05** | ***P* = 0.01** | ***P* = 0.001** | ***P* = 0.05** | ***P* = 0.01** | ***P* = 0.001** | ***P* = 0.05** | ***P* = 0.01** | ***P* = 0.001** |
| 0.5 | 4 | 100 | 100 | 100 | 100 | 100 | 99.9 | 100 | 99.8 | 98.9 | 99.9 | 99.6 | 98.0 |
|  | 3 | 100 | 100 | 100 | 99.9 | 99.7 | 98.0 | 99.4 | 97.6 | 90.8 | 99.0 | 96.1 | 87.1 |
|  | 2 | 100 | 99.5 | 96.6 | 93.4 | 81.7 | 58.7 | 84.6 | 66.4 | 39.8 | 81.1 | 61.3 | 34.6 |
|  | 1.5 | 86.0 | 67.9 | 40.2 | 54.1 | 30.5 | 11.3 | 42.6 | 21.3 | 6.7 | 39.5 | 19.0 | 5.7 |
| 0.4 | 4 | 100 | 100 | 100 | 100 | 100 | 99.9 | 100 | 99.9 | 99.4 | 99.9 | 99,8 | 98.8 |
|  | 3 | 100 | 100 | 100 | 100 | 99.8 | 98.7 | 99.7 | 98.3 | 92.4 | 99.4 | 97.1 | 88.8 |
|  | 2 | 99.9 | 99.5 | 97.2 | 93.9 | 82.6 | 59.3 | 85.2 | 66.6 | 39.1 | 81.7 | 61.3 | 33.6 |
|  | 1.5 | 86.0 | 67.9 | 40.2 | 53.7 | 29.8 | 10.7 | 41.8 | 20.3 | 6.1 | 38.5 | 18.0 | 5.1 |
| 0.3 | 4 | 100 | 100 | 100 | 100 | 100 | 99.9 | 100 | 99.9 | 99.4 | 99.9 | 99.8 | 98.8 |
|  | 3 | 100 | 100 | 100 | 100 | 99.8 | 98.4 | 99.6 | 98.1 | 91.1 | 99.3 | 96.7 | 86.9 |
|  | 2 | 99.9 | 99.3 | 96.0 | 92.5 | 79.3 | 53.9 | 82.6 | 61.8 | 33.4 | 78.7 | 56.2 | 28.2 |
|  | 1.5 | 82.9 | 63.1 | 35.3 | 49.5 | 26.1 | 8.7 | 37.8 | 17.2 | 4.7 | 34.7 | 15.1 | 3.9 |
| 0.2 | 4 | 100 | 100 | 100 | 100 | 100 | 99.9 | 100 | 99.9 | 98.7 | 99.9 | 99.7 | 97.4 |
|  | 3 | 100 | 100 | 100 | 99.9 | 99.4 | 96.1 | 99.1 | 95.8 | 83.3 | 98.5 | 93.4 | 77.0 |
|  | 2 | 99.5 | 97.7 | 90.3 | 86.8 | 68.4 | 40.1 | 74.0 | 49.3 | 22.0 | 69.4 | 43.8 | 17.9 |
|  | 1.5 | 73.9 | 51.0 | 24.6 | 40.6 | 19.1 | 5.5 | 30.2 | 12.1 | 2.8 | 27.5 | 10.6 | 2.3 |
| 0.1 | 4 | 100 | 100 | 100 | 100 | 99.7 | 98.0 | 99.6 | 97.5 | 88.1 | 99.2 | 95.8 | 82.0 |
|  | 3 | 100 | 100 | 99.7 | 98.3 | 93.1 | 77.4 | 93.4 | 79.2 | 50.8 | 90.7 | 73.1 | 42.4 |
|  | 2 | 94.8 | 84.8 | 63.3 | 66.3 | 41.1 | 16.6 | 50.6 | 25.3 | 7.4 | 46.0 | 21.4 | 5.7 |
|  | 1.5 | 51.7 | 28.5 | 10.2 | 25.3 | 9.5 | 2.0 | 18.3 | 5.9 | 1.0 | 16.6 | 5.1 | 1.0 |
| 0.05 | 4 | 100 | 100 | 99.7 | 98.4 | 93.7 | 79.1 | 93.6 | 79.5 | 50.8 | 90.9 | 73.0 | 41.4 |
|  | 3 | 99.5 | 97.8 | 91.5 | 86.7 | 68.0 | 39.5 | 72.2 | 45.3 | 17.8 | 66.8 | 38.6 | 13.2 |
|  | 2 | 76.6 | 55.5 | 29.4 | 41.3 | 19.1 | 5.3 | 28.6 | 10.5 | 2.0 | 25.4 | 8.6 | 1.5 |
|  | 1.5 | 31.7 | 13.9 | 3.7 | 1.5 | 4.6 | 1.0 | 10.8 | 2.8 | 0.0 | 9.8 | 2.5 | 0.0 |

Performed in excel using a script prepared in-house at CIMR by Dr Heather Cordell. The table shows the percent power for different alpha error rates (*P*=0.05, 0.01, 0.001) for SNPs with different minor allele frequencies (0.5 to 0.05) and different effect sizes (odds ratios 1.5 to 4). Yellow indicates the example of power highlighted in the main text methods section.
