# Supplementary material for: Genetic and Epigenetic Factors at COL2A1 and ABCA4 Influence Clinical Outcome in Congenital Toxoplasmosis
Source: PLoS One. 2008 Jun 4;3(6):e2285. doi: 10.1371/journal.pone.0002285 (PMC2390765; doi:10.1371/journal.pone.0002285)
Supplement: Table S3 — Power calculations for the NCCCTS cohort. (0.07 MB DOC) [file pone.0002285.s004.doc]

**Table S3.** Power calculations for the NCCCTS cohort.

| **SNP Allele Freq** | **Effect Size**  **(Odds Ratio)** | **124 Trios** | | | **113 Trios** | | | | **103 Trios** | | |
| --- | --- | --- | --- | --- | --- | --- | --- | --- | --- | --- | --- |
|  |  | ***P* = 0.05** | ***P* = 0.01** | ***P* = 0.001** | ***P* = 0.05** | ***P* = 0.01** | ***P* = 0.001** | ***P* = 0.05** | | ***P* = 0.01** | ***P* = 0.001** |
| 0.5 | 3 | 100 | 100 | 99.7 | 100 | 100 | 99.4 | 100 | | 99.9 | 98.5 |
|  | 2 | 98.5 | 92.5 | 73.8 | 97.7 | 90.0 | 68.3 | 96.2 | | 85.5 | 60.1 |
|  | 1.5 | 72.0 | 45.6 | 18.7 | 68.8 | 41.9 | 16.3 | 64.1 | | 37.0 | 13.4 |
| 0.4 | 3 | 100 | 99.9 | 99.1 | 100 | 99.9 | 98.3 | 99.9 | | 99.6 | 96.3 |
|  | 2 | 97.5 | 89.2 | 66.9 | 96.4 | 86.2 | 61.2 | 94.5 | | 81.0 | 53.0 |
|  | 1.5 | 69.0 | 42.1 | 16.4 | 65.7 | 38.6 | 14.3 | 61.1 | | 34.0 | 11.7 |
| 0.3 | 3 | 100 | 99.5 | 95.9 | 99.9 | 99.1 | 93.6 | 99.8 | | 98.2 | 89.1 |
|  | 2 | 94.7 | 81.6 | 53.9 | 93.0 | 77.7 | 48.3 | 90.1 | | 71.6 | 40.8 |
|  | 1.5 | 62.4 | 35.3 | 12.4 | 59.3 | 32.3 | 10.8 | 54.8 | | 28.4 | 8.9 |
| 0.2 | 3 | 99.4 | 96.0 | 81.5 | 99.0 | 94.2 | 76.3 | 98.2 | | 90.7 | 68.0 |
|  | 2 | 86.6 | 65.2 | 34.1 | 83.9 | 60.8 | 29.9 | 79.6 | | 54.5 | 24.6 |
|  | 1.5 | 51.3 | 25.4 | 7.5 | 48.5 | 23.2 | 6.6 | 44.6 | | 20.4 | 5.4 |
| 01 | 3 | 90.3 | 70.6 | 37.8 | 87.9 | 66.0 | 33.0 | 84.0 | | 59.2 | 26.8 |
|  | 2 | 63.4 | 35.5 | 12.1 | 60.1 | 32.4 | 10.4 | 55.5 | | 28.3 | 8.5 |
|  | 1.5 | 33.9 | 13.4 | 3.0 | 32.0 | 12.3 | 2.6 | 29.5 | | 10.9 | 2.2 |
| 0.05 | 3 | 65.7 | 36.1 | 11.4 | 62.2 | 32.7 | 9.7 | 57.3 | | 28.3 | 7.7 |
|  | 2 | 40.4 | 17.0 | 4.0 | 38.1 | 15.5 | 3.5 | 34.9 | | 13.6 | 2.9 |
|  | 1.5 | 22.2 | 7.2 | 1.3 | 21.1 | 6.7 | 1.2 | 19.6 | | 6.1 | 1.0 |

To determine the power of the NCCCTS cohort to detect allelic association, TDT power approximations were made using the method of Knapp [3]. The calculations compare the power of 124 (all affected children), 113 (all children with eye disease) and 103 (all children with brain disease) fully genotyped parent/offspring trios. Theoretical power to detect allelic association was made assuming a multiplicative model and for different effect sizes (odds ratios) using markers of different allele frequencies. Results are given as a first approximation of the percentage power to detect allelic association at alpha error rates (one-sided *P* values for replication) *P* = 0.05, *P* = 0.01 or *P* = 0.001. Yellow indicates the example of power highlighted in the main text methods section.
