# Supplementary material for: Genetic and Epigenetic Factors at COL2A1 and ABCA4 Influence Clinical Outcome in Congenital Toxoplasmosis
Source: PLoS One. 2008 Jun 4;3(6):e2285. doi: 10.1371/journal.pone.0002285 (PMC2390765; doi:10.1371/journal.pone.0002285)
Supplement: Table S4 — Gene specific primers for reverse transcription (A); and PCR primers used to obtain products for sequencing (B). (0.04 MB DOC) [file pone.0002285.s005.doc]

**Table S4.** Gene specific primers for reverse transcription (A); and PCR primers used to obtain products for sequencing (B).

(A)

| Gene | Sequence |
| --- | --- |
| COL2A1_GSP1 | ATCCTCTCTCACCACGTTGC |
| COL2A1_GSP2 | GCCTTGTTCACCTTTGAAGC |
| ABCA4_GSP1 | GAAGGTGCTGAGCAGAAAGC |

(B)

| Gene/SNP | Gel/  Sequence | Template | Sequence | Product Size |
| --- | --- | --- | --- | --- |
| *ABCA4*/  rs3112831 | Gel | cDNA –  (Both Isoforms) | F - ACCTGCAGCACGAAGGATAC | 248bp (Isoform +10)  131bp (Isoform -10) |
|  |  |  | R - GAGGGCCCTTGTAGAGGAAG |  |
|  | Sequence | cDNA –  (Isoform +10) | F - TCCTGTACACTCCTGATTCACC | 163bp |
|  |  |  | R - CTGATCATGTTCATCTGTGTGC |  |
|  |  | gDNA | F - CCAGGAAGTTGGGAGATGG | 467bp |
|  |  |  | R - CCACTTGGTGACTGCTTGG |  |
| *COL2A1*/  rs3737548 | Gel | cDNA –  (Both Isoforms) | F - GATTCGCCTCGGGGCTC | 358bp (Isoform IIA)  151bp (Isoform IIB) |
|  |  |  | R - TCCTTTGGGTCCTACAATATCC |  |
|  | Sequence | cDNA –  (Isoform IIA) | F - ACGTGAAAGACTGCCTCAGC | 392bp |
|  |  |  | R - TCCTTGCATTACTCCCAACTG |  |
|  |  | cDNA –  (Isoform IIB) | F - ATGTCCGGCAACCAGGAC | 317bp |
|  |  |  | R - TCCTTGCATTACTCCCAACTG |  |
|  |  | gDNA | F - GGCTCTTGGAGAAACACTGC | 415bp |
|  |  |  | R - TGGTACAGATGCCAGGAAGC |  |

All primers shown 5’ to 3’.
