# Supplementary material for: Genetic and Epigenetic Factors at COL2A1 and ABCA4 Influence Clinical Outcome in Congenital Toxoplasmosis
Source: PLoS One. 2008 Jun 4;3(6):e2285. doi: 10.1371/journal.pone.0002285 (PMC2390765; doi:10.1371/journal.pone.0002285)
Supplement: Table S5 — Intra-locus and inter-locus forward stepwise logistic regression analysis for allelic associations comparing mothers of affected children with mothers of unaffected children from the EMSCOT cohort. (0.07 MB DOC) [file pone.0002285.s006.doc]

**Table S5.** Intra-locus and inter-locus forward stepwise logistic regression analysis for allelic associations comparing mothers of affected children with mothers of unaffected children from the EMSCOT cohort.

| **Intra-locus stepwise analysis:** | | | | |
| --- | --- | --- | --- | --- |
| **Null Model** | **Alternative Model** | **χ^2^** | **df** | ***P*** |
| **Adding a SNP at *ABCA4*** | | | | |
| rs2297633 | rs2297633 + **rs1761375** | 1.55 | 1 | 0.2135 |
| rs2297633 | rs2297633 + **rs3112831** | 0.21 | 1 | 0.6476 |
| rs2297633 | rs2297633 + **rs952499** | 1.89 | 1 | 0.1691 |
| rs1761375 | rs1761375 + **rs2297633** | 2.91 | 1 | 0.088 |
| rs1761375 | rs1761375 + **rs3112831** | 0.00 | 1 | 0.9979 |
| rs1761375 | rs1761375 + **rs952499** | 0.74 | 1 | 0.3904 |
| rs3112831 | rs3112831+ **rs2297633** | 7.59 | 1 | **0.0048** |
| rs3112831 | rs3112831+ **rs1761375** | 6.13 | 1 | **0.0133** |
| rs3112831 | rs3112831+ **rs952499** | 1.68 | 1 | 0.1952 |
| rs952499 | rs952499 + **rs2297633** | 7.78 | 1 | **0.0053** |
| rs952499 | rs952499 + **rs1761375** | 6.78 | 1 | **0.0092** |
| rs952499 | rs952499 + **rs3112831** | 0.29 | 1 | 0.5905 |
|  |  |  |  |  |
| rs2297633 + rs1761375 | rs2297633 + rs1761375 + **rs952499** | 1.04 | 1 | 0.3072 |
| rs2297633 + rs1761375 | rs2297633 + rs1761375 + **rs3112831** | 0.09 | 1 | 0.7599 |
|  | | | | |
| **Adding a SNP at *COL2A1*** | | | | |
| rs2070739 | rs270739 + **rs2276455** | 6.54 | 1 | **0.0106** |
| rs2070739 | rs270739 + **rs1635544** | 4.46 | 1 | **0.0346** |
| rs2070739 | rs270739 + **rs3803183** | 9.00 | 1 | **0.0027** |
| rs2276455 | rs2276455 + **rs2070739** | 1.73 | 1 | 0.1878 |
| rs2276455 | rs2276455 + **rs1635544** | 1.63 | 1 | 0.2018 |
| rs2276455 | rs2276455 + **rs3803183** | 3.45 | 1 | 0.0631 |
| rs1635544 | rs1635544 + **rs2070739** | 0.4 | 1 | 0.5276 |
| rs1635544 | rs1635544 + **rs2276455** | 0.00 | 1 | 0.9854 |
| rs1635544 | rs1635544 + **rs3803183** | 0.14 | 1 | 0.7052 |
| rs3803183 | rs3803183 + **rs2070739** | 2.45 | 1 | 0.1175 |
| rs3803183 | rs3803183 + **rs2276455** | 1.88 | 1 | 0.1702 |
| rs3803183 | rs3803183 + **rs1635544** | 3.93 | 1 | **0.0474** |
|  |  |  |  |  |
| rs2276455 + rs1635544 | rs2276455 + rs1635544 + r**s3803183** | 0.13 | 1 | 0.7219 |
| rs2276455 + rs3803183 | rs2276455 + rs3803183 + **rs1635544** | 2.71 | 1 | 0.0998 |
| rs1635544 + rs3803183 | rs1635544 + rs3803183 + **rs2276455** | 0.47 | 1 | 0.4913 |
|  | | | | |
| rs2276455 + rs1635544 | rs2276455 + rs1635544 + r**s3803183** | 0.13 | 1 | 0.7219 |
| rs2276455 + rs3803183 | rs2276455 + rs3803183 + **rs1635544** | 2.71 | 1 | 0.0998 |
| rs1635544 + rs3803183 | rs1635544 + rs3803183 + **rs2276455** | 0.47 | 1 | 0.4913 |
| **Inter-locus stepwise analysis:** | | | | |
| **Null Model** | **Alternative Model** | **χ^2^** | **df** | ***P*** |
| **Adding a SNP at *COL2A1*** | | | | |
| *ABCA4*/rs1761375 | *ABCA4*/rs1761375 + ***COL2A1*/rs2276455** | 8.70 | 1 | **0.0032** |
| **Adding a SNP at *ABCA4*** | | | | |
| *COL2A1*/rs2276455 | *COL2A1*/rs2276455 + ***ABCA4*/rs1761375** | 5.22 | 1 | **0.0223** |

The intra-locus test determines whether multiple SNPs that show significant single point allelic associations within each candidate gene locus contribute independent main effects. A significant Wald χ^2^  test comparing null and alternative models indicates that the marker added (bold) under the alternative model is contributing an independent main effect from marker(s) considered under the null hypothesis. The inter-locus test determines whether SNPs at the two different loci contribute independent main effects. Significant *P* values (*P*≤0.05) are shown in bold. Similar results were obtained (data not shown) and conclusions drawn when the data for affected children were compared with unaffected children for both loci, when the data for mothers of children with eye lesions were compared with unaffected children for both loci, and for *ABCA4* when mothers of children with brain lesions were compared with mothers of unaffected children or children with brain lesions were compared with unaffected children. Comparisons were only made where mothers or children had complete genotype data for all markers included in the model.
