# Supplementary material for: Genetic and Epigenetic Factors at COL2A1 and ABCA4 Influence Clinical Outcome in Congenital Toxoplasmosis
Source: PLoS One. 2008 Jun 4;3(6):e2285. doi: 10.1371/journal.pone.0002285 (PMC2390765; doi:10.1371/journal.pone.0002285)
Supplement: Table S6 — Intra-locus and inter-locus forward stepwise conditional logistic regression analysis in the NCCCTS cohort. (0.07 MB DOC) [file pone.0002285.s007.doc]

**Table S6.**  Intra-locus and inter-locus forward stepwise conditional logistic regression analysis in the NCCCTS cohort.

| **Intra-locus stepwise analysis:** | | | | |
| --- | --- | --- | --- | --- |
| **Null Model** | **Alternative Model** | **χ^2^** | **df** | ***P*** |
| **Adding a SNP at *COL2A1*** | | | | |
| rs6823 | rs6823 + **rs2070739** | 3.79 | 1 | **0.0514** |
| rs6823 | rs6823+ **rs2276455** | 0.94 | 1 | 0.3332 |
| rs6823 | rs6823+ **rs2276454** | 3.53 | 1 | 0.0604 |
| rs6823 | rs6823 + **rs1635544** | 2.44 | 1 | 0.1180 |
| rs6823 | rs6823+ **rs3803183** | 2.23 | 1 | 0.1353 |
| rs2070739 | rs2070739 **+ rs6823** | **4.42** | **1** | **0.0354** |
| rs2070739 | rs270739 + **rs2276455** | 2.77 | 1 | 0.0963 |
| rs2070739 | rs270739 + **rs2276454** | **4.23** | **1** | **0.0396** |
| rs2070739 | rs270739 + **rs1635544** | **4.10** | **1** | **0.0429** |
| rs2070739 | rs270739 + **rs3803183** | 1.38 | 1 | 0.2407 |
| rs2276455 | rs2276455 + **rs6823** | **5.52** | **1** | **0.0188** |
| rs2276455 | rs2276455 + **rs2070739** | 0.00 | 1 | 0.9893 |
| rs2276455 | rs2276455 **+ rs2276454** | NA | NA | NA |
| rs2276455 | rs2276455 + **rs1635544** | **4.11** | **1** | **0.0427** |
| rs2276455 | rs2276455 + **rs3803183** | 0.36 | 1 | 0.5481 |
| rs2276454 | rs2276454 + **rs6823** | 0.58 | 1 | 0.4472 |
| rs2276454 | rs2276454 + **rs2070739** | 0.01 | 1 | 0.9223 |
| rs2276454 | rs2276454 + **rs2276455** | NA | NA | NA |
| rs2276454 | rs2276454 + **rs1635544** | 1.28 | 1 | 0.2576 |
| rs2276454 | rs2276454 + **rs3803183** | 0.12 | 1 | 0.7304 |
| rs1635544 | rs1635544 + **rs6823** | 1.33 | 1 | 0.2484 |
| rs1635544 | rs1635544 + **rs2070739** | 0.08 | 1 | 0.7770 |
| rs1635544 | rs1635544 + **rs2276455** | 0.51 | 1 | 0.4739 |
| rs1635544 | rs1635544 + **rs2276454** | 0.04 | 1 | 0.8478 |
| rs1635544 | rs1635544 + **rs3803183** | 0.65 | 1 | 0.4188 |
| rs3803183 | rs3803183 + **rs6823** | 1.42 | 1 | 0.2336 |
| rs3803183 | rs3803183 + **rs2070739** | 0.06 | 1 | 0.8001 |
| rs3803183 | rs3803183 + **rs2276455** | 1.87 | 1 | 0.1713 |
| rs3803183 | rs3803183 + **rs2276454** | 2.73 | 1 | 0.0985 |
| rs3803183 | rs3803183 + **rs1635544** | 2.02 | 1 | 0.1555 |
| **Inter-locus stepwise analysis:** | | | | |
| **Null Model** | **Alternative Model** | **χ^2^** | **df** | ***P*** |
| **Adding a SNP at *COL2A1*** | | | | |
| *ABCA4*/rs952499 | *ABCA4*/rs952499 + ***COL2A1*/rs1635544** | **5.14** | **1** | **0.0233** |
| **Adding a SNP at *ABCA4*** | | | | |
| *COL2A1***/**rs1635544 | *COL2A1*/rs1635544 + ***ABCA4*/rs952499** | 4.19 | 2 | 0.1229 |

Conditional stepwise logistic regression analysis was performed on the NCCCTS cohort using a case/pseudocontrol data set generated from the affected child/parent trios. Only trios with complete genotype data for all markers included in the model were used in the analysis. The intra-locus test determines whether multiple SNPs that show significant single point allelic associations within each candidate gene locus contribute independent main effects. A significant Wald χ^2^  test comparing null and alternative models indicates that the marker added (bold) under the alternative model is contributing an independent main effect from marker(s) considered under the null hypothesis. The inter-locus test determines whether SNPs at the two different loci contribute independent main effects. Significant *P* values (*P*≤0.05) are shown in bold. Similar results were obtained (data not shown) and conclusions drawn when the data were analysed for the eye lesion phenotype for *COL2A1*.
