# Supplementary material for: Genetic and Epigenetic Factors at COL2A1 and ABCA4 Influence Clinical Outcome in Congenital Toxoplasmosis
Source: PLoS One. 2008 Jun 4;3(6):e2285. doi: 10.1371/journal.pone.0002285 (PMC2390765; doi:10.1371/journal.pone.0002285)
Supplement: Table S7 — Summary of haplotype associations across COL2A1 analysed using TRANSMIT for the NCCCTS cohort. (0.07 MB DOC) [file pone.0002285.s008.doc]

**Table S7.** Summary of haplotype associations across COL2A1 analysed using TRANSMIT for the NCCCTS cohort.

|  | Haplotype | Frequency | rs6823 | rs2070739 | rs2276455 | rs2276454 | rs1635544 | rs1793958 | rs3803183 |
| --- | --- | --- | --- | --- | --- | --- | --- | --- | --- |
| Over transmitted | 2.1 | 0.14 | χ^2^ = 3.20; *P* = 0.074 | |  |  |  |  |  |
|  | 1.2 | 0.14 |  | χ^2^ = 1.39; *P* = 0.238 | |  |  |  |  |
|  | 2.1 | 0.62 |  |  | χ^2^ = 2.31; *P* = 0.128 | |  |  |  |
|  | 1.1 | 0.54 |  |  |  | χ^2^ = 2.92; ***P* = 0.048** | |  |  |
|  | 1.1 | 0.34 |  |  |  |  | χ^2^ = 3.41; *P* = 0.065 | |  |
|  | 2.1 | 0.22 |  |  |  |  |  | χ^2^ = 0.66; *P* = 0.417 | |
|  | 2.1.2 | 0.13 | χ^2^ = 1.54; *P* = 0.215 | | |  |  |  |  |
|  | 1.2.1 | 0.15 |  | χ^2^ = 1.37; *P* = 0.242 | | |  |  |  |
|  | 2.1.1 | 0.54 |  |  | χ^2^ = 3.52; *P* = 0.061 | | |  |  |
|  | 1.1.1 | 0.37 |  |  |  | χ^2^ = 4.12; ***P* = 0.042** | | |  |
|  | 1.1.2 | 0.34 |  |  |  |  | χ^2^ = 3.29; *P* = 0.070 | | |
|  |  |  |  |  |  |  |  |  |  |
| Under transmitted | 2.2 | 0.43 | χ^2^ = 5.53; ***P* = 0.021** | |  |  |  |  |  |
|  | 2.1 | 0.38 |  | χ^2^ = 5.59; ***P* = 0.018** | |  |  |  |  |
|  | 1.2 | 0.38 |  |  | χ^2^ = 1.90; *P* = 0.168 | |  |  |  |
|  | 2.2 | 0.38 |  |  |  | χ^2^ = 4.49; ***P* = 0.034** | |  |  |
|  | 2.1 | 0.22 |  |  |  |  | χ^2^ = 4.26; *P* = **0.039** | |  |
|  | 1.2 | 0.55 |  |  |  |  |  | χ^2^ = 0.02; *P* = 0.887 | |
|  | 2.2.1 | 0.33 | χ^2^ = 6.70; ***P* = 0.009** | | |  |  |  |  |
|  | 2.1.2 | 0.37 |  | χ^2^ = 4.08; ***P* = 0.043** | | |  |  |  |
|  | 1.2.2 | 0.37 |  |  | χ^2^ = 4.04; ***P* = 0.044** | | |  |  |
|  | 2.2.1 | 0.15 |  |  |  | χ^2^ = 4.81; ***P* = 0.028** | | |  |
|  | 2.1.2 | 0.21 |  |  |  |  | χ^2^ = 4.14; ***P* = 0.042** | | |
|  | 2.2.1.2.2.1.2 |  | χ^2^ = 8.29; ***P* = 0.004** | | | | | | |

Haplotype associations are shown for common (frequency >0.1) pair-wise and triple combinations of SNPs across *COL2A1*. One degree of freedom χ^2^ tests determine whether the haplotype indicated was over transmitted or under transmitted from heterozygous parents to affected offspring. Bold indicates *P*≤0.05. The yellow filled boxes indicate a break in the haplotype associations across *COL2A1* within the main linkage disequilibrium block (**Figure 1, main text**).
