# Supplementary material for: Genetic and Epigenetic Factors at COL2A1 and ABCA4 Influence Clinical Outcome in Congenital Toxoplasmosis
Source: PLoS One. 2008 Jun 4;3(6):e2285. doi: 10.1371/journal.pone.0002285 (PMC2390765; doi:10.1371/journal.pone.0002285)
Supplement: Table S8 — Absolute numbers of individuals with each genotype at each marker according clinical to phenotype and appropriate control group for (A) children and (B) mothers in the EMSCOT study. (0.19 MB DOC) [file pone.0002285.s009.doc]

**Table S8.** Shows absolute numbers of individuals with each genotype at each marker according clinical to phenotype and appropriate control group for (A) children and (B) mothers in the EMSCOT study.

(A)

| **Marker** | **Genotype** | **Children** | | | | | | | |
| --- | --- | --- | --- | --- | --- | --- | --- | --- | --- |
|  |  | **Un-infected** | **Infected** | **Un-affected** | **Affected** | **No Eye** | **Eye** | **No Brain** | **Brain** |
| COL2A1_rs6823 | G/G | 39 | 45 | 29 | 16 | 35 | 10 | 38 | 7 |
|  | G/C | 78 | 75 | 46 | 29 | 58 | 17 | 57 | 28 |
|  | C/C | 68 | 70 | 51 | 19 | 56 | 14 | 58 | 22 |
| COL2A1_rs2070739 | T/T | 2 | 3 | 2 | 1 | 3 | 0 | 2 | 1 |
|  | T/C | 35 | 24 | 16 | 8 | 18 | 6 | 20 | 4 |
|  | C/C | 173 | 188 | 122 | 66 | 145 | 43 | 148 | 40 |
| COL2A1_rs2276455 | A/A | 33 | 37 | 31 | 6 | 31 | 6 | 33 | 4 |
|  | A/G | 74 | 76 | 48 | 28 | 59 | 17 | 60 | 16 |
|  | G/G | 75 | 78 | 48 | 30 | 58 | 21 | 61 | 17 |
| COL2A1_rs2276454 | G/G | 81 | 82 | 53 | 29 | 61 | 21 | 67 | 15 |
|  | G/A | 90 | 84 | 53 | 31 | 67 | 17 | 66 | 18 |
|  | A/A | 35 | 40 | 32 | 8 | 32 | 8 | 34 | 6 |
| COL2A_rs1635544 | T/T | 38 | 49 | 32 | 17 | 39 | 10 | 35 | 14 |
|  | T/C | 57 | 62 | 41 | 21 | 48 | 14 | 52 | 10 |
|  | C/C | 26 | 33 | 25 | 8 | 26 | 7 | 30 | 3 |
| COL2A1_rs1793958 | G/G | 79 | 88 | 55 | 33 | 64 | 24 | 71 | 17 |
|  | G/A | 81 | 77 | 54 | 23 | 63 | 14 | 62 | 15 |
|  | A/A | 35 | 36 | 27 | 9 | 32 | 4 | 29 | 7 |
| COL2A1_rs3803183 | T/T | 7 | 3 | 2 | 1 | 3 | 0 | 3 | 1 |
|  | T/A | 56 | 40 | 24 | 16 | 29 | 11 | 32 | 8 |
|  | A/A | 145 | 168 | 116 | 52 | 132 | 36 | 138 | 30 |
| ABCR_rs1801574 | G/G | 11 | 9 | 6 | 3 | 6 | 3 | 9 | 0 |
|  | G.C | 67 | 74 | 50 | 24 | 60 | 14 | 57 | 17 |
|  | C/C | 132 | 121 | 79 | 42 | 91 | 30 | 97 | 24 |
| ABCR_rs2275033 | T/T | 31 | 39 | 27 | 12 | 30 | 9 | 34 | 5 |
|  | T/C | 92 | 98 | 66 | 32 | 78 | 20 | 79 | 19 |
|  | C/C | 75 | 69 | 43 | 26 | 52 | 17 | 54 | 15 |
| ABCR_rs2297671 | G/G | 54 | 58 | 35 | 23 | 42 | 16 | 43 | 15 |
|  | G/A | 99 | 86 | 60 | 26 | 69 | 17 | 73 | 13 |
|  | A/A | 49 | 49 | 30 | 19 | 37 | 12 | 39 | 10 |
| ABCR_rs2297633 | T/T | 24 | 22 | 21 | 1 | 22 | 0 | 21 | 1 |
|  | T/G | 62 | 64 | 42 | 22 | 48 | 16 | 50 | 14 |
|  | G/G | 110 | 112 | 70 | 42 | 84 | 28 | 88 | 24 |
| ABCR_rs1761375 | G/G | 116 | 115 | 73 | 42 | 85 | 30 | 91 | 24 |
|  | G/A | 69 | 69 | 47 | 22 | 56 | 13 | 56 | 13 |
|  | A/A | 24 | 24 | 19 | 5 | 21 | 3 | 21 | 3 |
| ABCR_rs3112831 | T/T | 106 | 103 | 65 | 38 | 79 | 24 | 80 | 23 |
|  | T/C | 60 | 68 | 43 | 25 | 48 | 20 | 57 | 11 |
|  | C/C | 29 | 30 | 23 | 7 | 28 | 2 | 24 | 6 |
| ABCR_rs952499 | T/T | 66 | 65 | 40 | 25 | 51 | 14 | 50 | 15 |
|  | T/C | 90 | 85 | 57 | 28 | 61 | 24 | 72 | 13 |
|  | C/C | 55 | 57 | 43 | 14 | 50 | 7 | 47 | 10 |

**…/ part (B)**

**(B**)

| **Marker** | **Genotype** | **Mothers** | | | | | | | |
| --- | --- | --- | --- | --- | --- | --- | --- | --- | --- |
|  |  | **Un-infected** | **Infected** | **Un-affected** | **Affected** | **No Eye** | **Eye** | **No Brain** | **Brain** |
| COL2A1_rs6823 | G/G | 41 | 44 | 29 | 15 | 35 | 9 | 36 | 8 |
|  | G/C | 90 | 76 | 49 | 27 | 62 | 14 | 57 | 19 |
|  | C/C | 64 | 86 | 66 | 20 | 71 | 15 | 75 | 11 |
| COL2A1_rs2070739 | T/T | 1 | 2 | 1 | 1 | 1 | 1 | 2 | 0 |
|  | T/C | 32 | 38 | 23 | 15 | 27 | 11 | 30 | 8 |
|  | C/C | 179 | 190 | 136 | 54 | 157 | 33 | 159 | 31 |
| COL2A1_rs2276455 | A/A | 45 | 45 | 37 | 8 | 41 | 4 | 40 | 5 |
|  | A/G | 80 | 76 | 56 | 20 | 60 | 16 | 60 | 16 |
|  | G/G | 66 | 65 | 39 | 26 | 51 | 14 | 52 | 13 |
| COL2A1_rs2276454 | G/G | 70 | 70 | 46 | 24 | 57 | 13 | 58 | 12 |
|  | G/A | 86 | 91 | 62 | 29 | 69 | 22 | 73 | 18 |
|  | A/A | 45 | 47 | 37 | 10 | 41 | 6 | 40 | 7 |
| COL2A_rs1635544 | T/T | 48 | 42 | 26 | 16 | 32 | 10 | 32 | 10 |
|  | T/C | 48 | 38 | 31 | 7 | 32 | 6 | 33 | 5 |
|  | C/C | 44 | 39 | 32 | 7 | 37 | 2 | 34 | 5 |
| COL2A1_rs1793958 | G/G | 93 | 79 | 55 | 24 | 62 | 17 | 63 | 16 |
|  | G/A | 81 | 91 | 60 | 31 | 71 | 20 | 76 | 15 |
|  | A/A | 35 | 41 | 31 | 10 | 35 | 6 | 34 | 7 |
| COL2A1_rs3803183 | T/T | 3 | 3 | 1 | 2 | 2 | 1 | 2 | 1 |
|  | T/A | 55 | 47 | 25 | 22 | 33 | 14 | 36 | 11 |
|  | A/A | 153 | 170 | 126 | 44 | 141 | 29 | 143 | 27 |
| ABCR_rs1801574 | G/G | 9 | 11 | 8 | 3 | 9 | 2 | 10 | 1 |
|  | G.C | 68 | 70 | 45 | 25 | 56 | 14 | 53 | 17 |
|  | C/C | 126 | 129 | 94 | 35 | 105 | 24 | 108 | 21 |
| ABCR_rs2275033 | T/T | 38 | 38 | 29 | 9 | 32 | 6 | 32 | 6 |
|  | T/C | 74 | 89 | 65 | 24 | 73 | 16 | 75 | 14 |
|  | C/C | 85 | 84 | 55 | 29 | 65 | 19 | 68 | 16 |
| ABCR_rs2297671 | G/G | 66 | 79 | 57 | 22 | 64 | 15 | 66 | 13 |
|  | G/A | 78 | 82 | 50 | 32 | 63 | 19 | 65 | 17 |
|  | A/A | 57 | 51 | 37 | 14 | 41 | 10 | 42 | 9 |
| ABCR_rs2297633 | T/T | 22 | 21 | 19 | 2 | 21 | 0 | 19 | 2 |
|  | T/G | 73 | 66 | 49 | 17 | 55 | 11 | 58 | 8 |
|  | G/G | 111 | 109 | 66 | 43 | 80 | 29 | 83 | 26 |
| ABCR_rs1761375 | G/G | 112 | 113 | 71 | 42 | 86 | 27 | 86 | 27 |
|  | G/A | 75 | 81 | 62 | 19 | 68 | 13 | 72 | 9 |
|  | A/A | 20 | 27 | 24 | 3 | 26 | 1 | 25 | 2 |
| ABCR_rs3112831 | T/T | 109 | 105 | 66 | 39 | 79 | 26 | 81 | 24 |
|  | T/C | 51 | 75 | 53 | 22 | 60 | 15 | 64 | 11 |
|  | C/C | 28 | 30 | 23 | 7 | 28 | 2 | 25 | 5 |
| ABCR_rs952499 | T/T | 68 | 55 | 33 | 22 | 42 | 13 | 40 | 15 |
|  | T/C | 82 | 97 | 68 | 29 | 74 | 23 | 83 | 14 |
|  | C/C | 57 | 62 | 48 | 14 | 55 | 7 | 54 | 8 |
