# Supplementary material for: Genetic and Epigenetic Factors at COL2A1 and ABCA4 Influence Clinical Outcome in Congenital Toxoplasmosis
Source: PLoS One. 2008 Jun 4;3(6):e2285. doi: 10.1371/journal.pone.0002285 (PMC2390765; doi:10.1371/journal.pone.0002285)
Supplement: Table S9 — Absolute numbers of individuals with each genotype at each marker according to clinical phenotype for the 124 (113 and 103) possible children included in the genetic study for the NCCCTS study. (0.09 MB DOC) [file pone.0002285.s010.doc]

**Table S9.** Shows absolute numbers of individuals with each genotype at each marker according to clinical phenotype for the 124 (113 and 103) possible children included in the genetic study for the NCCCTS study.

| **Marker** | **Genotype** | **Affected** | **Eye** | **Brain** |
| --- | --- | --- | --- | --- |
| COL2A1_rs6823 | G/G | 19 | 18 | 14 |
|  | G/C | 69 | 65 | 57 |
|  | C/C | 32 | 28 | 26 |
| COL2A1_rs2070739 | T/T | 3 | 3 | 3 |
|  | T/C | 42 | 35 | 37 |
|  | C/C | 77 | 74 | 59 |
| COL2A1_rs2276455 | A/A | 12 | 12 | 9 |
|  | A/G | 49 | 42 | 38 |
|  | G/G | 45 | 42 | 38 |
| COL2A1_rs2276454 | G/G | 53 | 50 | 45 |
|  | G/A | 53 | 46 | 42 |
|  | A/A | 13 | 13 | 10 |
| COL2A_rs1635544 | T/T | 43 | 40 | 36 |
|  | T/C | 62 | 55 | 50 |
|  | C/C | 17 | 17 | 13 |
| COL2A1_rs1793958 | G/G | 32 | 30 | 24 |
|  | G/A | 52 | 50 | 44 |
|  | A/A | 21 | 16 | 16 |
| COL2A1_rs3803183 | T/T | 5 | 3 | 5 |
|  | T/A | 50 | 45 | 43 |
|  | A/A | 65 | 62 | 50 |
| ABCR_rs1801574 | G/G | 8 | 8 | 8 |
|  | G.C | 29 | 29 | 23 |
|  | C/C | 82 | 72 | 65 |
| ABCR_rs2275033 | T/T | 14 | 12 | 13 |
|  | T/C | 51 | 47 | 40 |
|  | C/C | 53 | 49 | 42 |
| ABCR_rs2297671 | G/G | 38 | 35 | 29 |
|  | G/A | 64 | 60 | 53 |
|  | A/A | 14 | 13 | 12 |
| ABCR_rs2297633 | T/T | 9 | 9 | 7 |
|  | T/G | 41 | 39 | 31 |
|  | G/G | 66 | 58 | 57 |
| ABCR_rs1761375 | G/G | 69 | 62 | 61 |
|  | G/A | 39 | 37 | 30 |
|  | A/A | 5 | 5 | 3 |
| ABCR_rs3112831 | T/T | 54 | 49 | 47 |
|  | T/C | 43 | 40 | 33 |
|  | C/C | 11 | 11 | 8 |
| ABCR_rs952499 | T/T | 31 | 28 | 26 |
|  | T/C | 58 | 54 | 48 |
|  | C/C | 30 | 29 | 22 |
